# Common genetic variants in complement genes other than *CFH*, *CD46* and the *CFHRs* are not associated with aHUS

**DOI:** 10.1016/j.molimm.2011.11.003

**Published:** 2012-01

**Authors:** Luca Ermini, Timothy H.J. Goodship, Lisa Strain, Michael E. Weale, Steven H. Sacks, Heather J. Cordell, Veronique Fremeaux-Bacchi, Neil S. Sheerin

**Affiliations:** a Institute of Cellular Medicine, Newcastle University, Framlington Place, Newcastle upon Tyne NE2 4HH, United Kingdom; b Institute of Genetic Medicine, Newcastle University, Central Parkway, Newcastle upon Tyne NE2 4HH, United Kingdom; c Northern Molecular Genetics Service, Newcastle upon Tyne Hospitals NHS Foundation Trust, United Kingdom; d Department of Medical and Molecular Genetics, King's College London, Guy's Hospital, London SE1 9RT, United Kingdom; e MRC Centre for Transplantation, King's College London, School of Medicine at Guy's, King's and St. Thomas’ Hospitals, London, United Kingdom; f Assistance Publique-Hopitaux de Paris, Hôpital Européen Georges-Pompidou, Service d‘Immunologie Biologique, Paris, France

**Keywords:** aHUS, Complement, Genetic polymorphisms

## Abstract

It is well established that common genetic variants in *CFH*, *CD46* and the *CFHRs* are additional risk factors for the development of aHUS. To examine the hypothesis that common variants in other complement genes have a similar effect we genotyped 501 SNPs in 47 complement genes in 94 aHUS patients from Newcastle, 126 aHUS patients from Paris, 374 UK controls and 165 French controls. We replicated the associations in *CFH*, *CD46* and the *CFHRs* but found no association with any other complement gene. The strongest associations replicated in both cohorts were found for four SNPs within *CD46* (*p*-value < 10^−3^) and five SNPs within *CFH* (*p*-value <5 × 10^−3^). Significant replicable associations with single SNPs in *CFHR2*, *CFHR4* and an intergenic SNP (*CR1–CD46*) were also found. Analysis of the Paris cohort showed that the association with *CD46* SNPs was only present in those patients with complement mutations. Haplotype analysis showed at-risk and protective haplotypes in both *CD46* and *CFH*. The *CD46* haplotype was only disease-associated in those patients with mutations.

## Highlights

► Hypothesis tested: common sequence variants in complement are associated with aHUS. ► SNPs in complement genes were genotyped in two independent aHUS cohorts. ► Only SNPs within *CFH*, *CD46* and the *CFHRs* showed a replicable association with aHUS. ► Complement regulators variability has the main role in determining the disease.

## 1 Introduction

Atypical hemolytic uremic syndrome (aHUS) is a disease characterised by excessive complement activation in the microvasculature (Noris and Remuzzi, 2009). Inherited and acquired abnormalities affecting components of the alternative complement pathway are found in ∼70% of patients (Noris et al., 2010). These include mutations in the genes encoding both complement regulators (factor H (Warwicker et al., 1998; Caprioli et al., 2001; Perez-Caballero et al., 2001; Richards et al., 2001; Sanchez-Corral et al., 2002; Venables et al., 2006), factor I (Fremeaux-Bacchi et al., 2004; Kavanagh et al., 2005, 2008; Caprioli et al., 2006; Nilsson et al., 2010), membrane cofactor protein (Noris et al., 2003; Richards et al., 2003) and thrombomodulin (Delvaeye et al., 2009)) and activators (factor B (Goicoechea de Jorge et al., 2007) and C3 (Fremeaux-Bacchi et al., 2008)); and autoantibodies against factor H (Moore et al., 2010). The penetrance of aHUS in the familial form of the disease is ∼60% (Caprioli et al., 2006). This is because multiple hits are necessary for the disease to manifest including a trigger, mutations (rare genetic variant) and at-risk haplotypes (common genetic variant) in complement genes. To date common genetic variants in *CFH*, *CD46* and the *CFHRs* (which all lie with the regulators of complement activation – RCA gene cluster at 1q32) have been reported to be risk factors for the development of aHUS (Caprioli et al., 2003; Esparza-Gordillo et al., 2005; Fremeaux-Bacchi et al., 2005; Pickering et al., 2007; Abarrategui-Garrido et al., 2009). In this study we have examined the hypothesis that common genetic variants in other complement genes apart from these have a similar effect. To do this we have genotyped tagged single nucleotide polymorphisms (SNPs) in 47 complement genes in two cohorts of aHUS patients. We have not found a significant association in any other complement gene apart from *CFH*, *CD46* and the *CFHRs*.

## 2 Methods

### 2.1 Patient and control cohorts

Atypical HUS was diagnosed clinically within two unrelated cohorts. The study was approved by the Northern and Yorkshire Multi-Centre Research Ethics Committee and informed consent obtained. The first cohort of patients comprised 94 aHUS cases from Newcastle upon Tyne, United Kingdom while the second included 126 aHUS patients from Paris, France. Clinical and demographic details of the subjects were recorded within two different databases. Mutation screening was performed on patients in local clinical laboratories.

We genotyped 374 DNA samples from healthy individuals within the Wellcome Trust Case Control Consortium (Burton et al., 2007; WTCCC, 2007) as a control population for the Newcastle cohort. As a control for the French aHUS cohort DNA samples from 165 healthy controls from France were used.

### 2.2 SNP selection and genotyping

We selected 501 SNPs within 47 complement genes (Supplementary Table 1). For tagging purposes, we clustered together genes with inter-gene distances less than 200 kb and used a hybrid tagging-plus-putative-function selection strategy similar in design to Cavalleri et al. (2007). This strategy allowed us to maximise the efficiency of SNP coverage. First, using TAMAL v2 program (Hemminger et al., 2006) SNPs with supposed functional effect and with a minor allele frequency (MAF) greater than 5% in the HapMap Phase 2 European-ancestry sample were chosen. SNPs were also selected if they met at least one of the following criteria: (1) non-synonymous coding SNP; (2) SNP within predicted promoter region; (3) SNP within evolutionarily conserved region; (4) SNP within predicted transcription factor binding site; (5) SNP within conserved miRNA target; (6) SNP within a splice region. In addition, 106 putative functional SNPs were added following a literature search.

All selected SNPs were tested for linkage disequilibrium using the Tagger program (de Bakker et al., 2005) and further SNPs were chosen to ensure an *r*^2^ greater than 0.9. Genotyping was performed by Sequenom using the iPlex platform, which utilises a multiplex PCR system followed by a single base pair extension step. The primer extension products are then analysed by MALDI-TOF mass spectrometry.

### 2.3 Statistical analysis

All statistical analyses were done with Plink Software version 1.05 (http://pngu.mgh.harvard.edu/∼purcell/plink/) and R statistical Software version 2.11 (http://www.r-project.org/). Linkage disequilibrium patterns between the SNPs were analysed using the Software HAPLOVIEW (Barrett et al., 2005). Genotyped SNPs and DNA samples were subjected to quality control (QC) procedures.

Sample duplication was assessed by estimation of pair wise identity by descent in PLINK (Pi-hat statistic). If a duplicate pair was detected (Pi-hat bigger than 0.9) both samples were excluded. Samples with a genotyping call rate below 95% were excluded as were SNPs with a missing genotype rate more than 5% and SNPs with an exact-test *p*-value for departure from Hardy–Weinberg equilibrium in controls with a *p*-value < 10^−3^. SNPs with a minor allele frequency less than 0.02 in our data set were also excluded.

All SNPs and samples that passed the quality control steps were assessed for association analysis. Statistical analysis on the Newcastle and Paris datasets was performed separately, and then data combined for a stratified analysis. A further statistical analysis was performed only in the Paris cohort for cases with and without complement gene mutations. The Cochran–Armitage trend test was used to establish genetic association in each cohort. Stratified analysis was calculated using the Cochran–Mantel–Haenszel (CMH) test. A Breslow–Day test was carried out to assess homogeneity of the common odds ratio across the two cohorts generated by the CMH test. Centre of origin (Newcastle/Paris) was added as a covariate in the stratified analyses. Haplotype-based association testing was performed using the --hap-logistic option in Plink. This option allows the estimation of an Odds Ratio for each haplotype relative to all other haplotypes combined.

## 3 Results

### 3.1 Analysis of cohort genotype data

We undertook a candidate genes analysis of 501 SNPs in 47 genes of the complement cascade. We genotyped 220 aHUS patients and 539 controls. After stringent quality control filtering, 444 SNPs within 419 samples (82 cases, 337 controls) in the Newcastle dataset and 461 SNPs within 251 (111 cases, 140 controls) samples in the Paris dataset were analysed. The association between each SNP and aHUS risk was tested using the Cochran–Armitage trend test and possible population stratification was corrected using genomic control (Devlin et al., 2001). The genomic inflation factor for the trend test was calculated for both cohorts separately (*λ*_GC_ = 1.28 for Newcastle cohort; *λ*_GC_ = 1.24 for Paris cohort).

### 3.2 Complement SNP analysis in aHUS cohorts

Single marker association analysis in the Newcastle and Paris cohorts was performed on the two datasets separately. When common SNPs typed in both cohorts were compared only SNPs located in or close to *CD46*, *CFH* and the *CFHRs* showed a replicable association with aHUS.

*CD46* showed a stronger association with aHUS than *CFH*. Four out of eight SNPs genotyped (rs2796275, rs2796278, rs10449303 and rs7144) displayed evidence of association with a *p*-value of < 10^−3^ in both cohorts.

We genotyped twenty SNPs within *CFH*, and five (rs1329423, rs12405238, rs3753396, rs424535, and rs1065489) showed an allelic association with aHUS with a *p*-value < 5 × 10^−3^ for the Newcastle cohort and a *p*-value <5 × 10^−5^ for the Paris Cohort.

In addition we found a common association for a marker in *CFHR2* (rs9427934, *p*-value = 4.24 × 10^−3^ in Newcastle and *p*-value = 5.54 × 10^−5^ in Paris cohorts) and another in *CFHR4* (rs3795341, *p*-value = 2.13 × 10^−3^ in Newcastle and *p*-value = 1.52 × 10^−5^ in Paris cohorts).

**Fig. 1:**
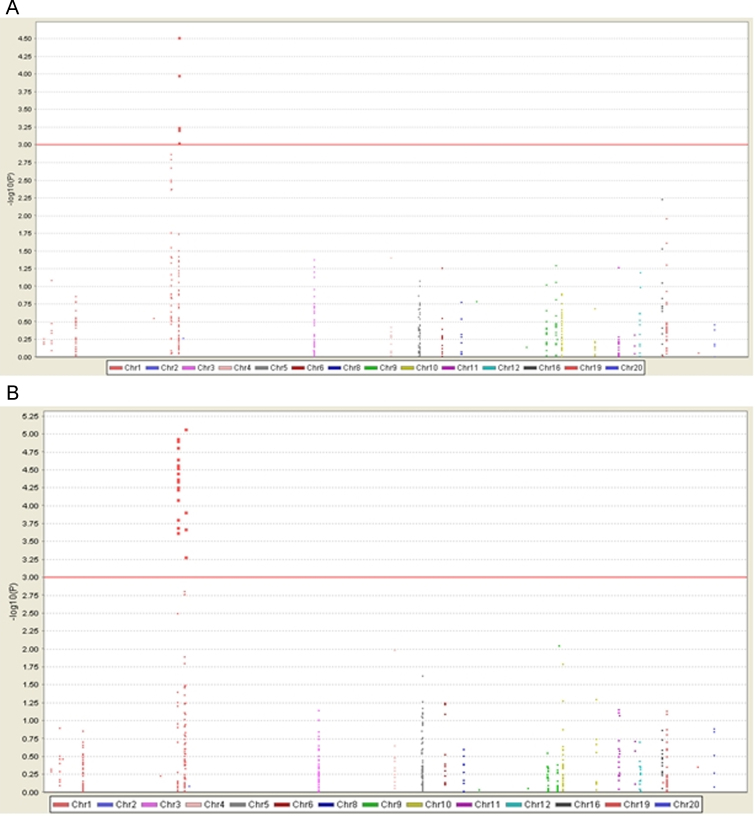
Manhattan plots of complement gene association in the two aHUS cohorts. Manhattan plots for the Newcastle (A) and Paris (B) cohorts are shown. *P*-values (−log_10_*p*-value, *y* axis) are plotted against their respective chromosomal positions (*x* axis). Each chromosome is shown in a different colour. The predefined level of significance, at 10^−3^ is shown with a horizontal red line. (For interpretation of the references to color in this figure legend, the reader is referred to the web version of this article.)

The most significant association, however was found for a SNP mapping to an intergenic position on chromosome 1 between *CR1* and *CD46* (rs2761434, *p*-value of 3.05 × 10^−5^ for the Newcastle cohort and a *p*-value of 8.53 × 10^−6^ for the Paris cohort). A Manhattan plot for the association study confirmed multiple associations at chromosome 1 in both cohorts (Fig. 1). The complete analysis of the Newcastle and Paris cohorts is provided in Supplementary Tables 2 and 3 respectively.

**Fig. 2:**
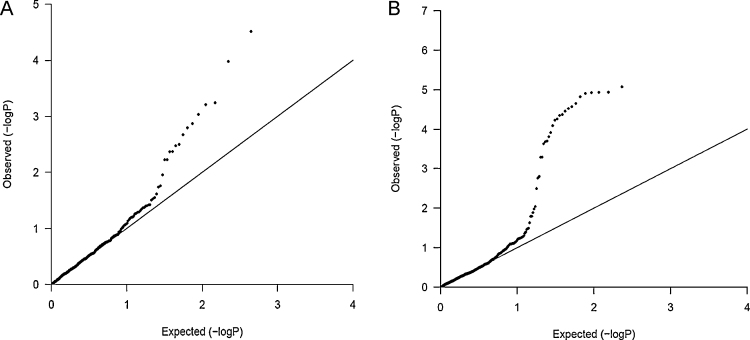
Quantile–quantile plots of Newcastle and Paris SNPs. The quantile–quantile plots of trend test observed versus expected *p*-values (−log_10_*P*) between (A) Newcastle cohort (*λ*_GC_ = 1.28) and (B) Paris cohort (*λ*_GC_ = 1.24). The straight line represents the null hypothesis of no true association.

A quantile–quantile plot of adjusted observed *p*-values for association between cases and controls showed a remarkable deviation from the null distribution within both cohorts (Fig. 2), which could be ascribed to the strong association observed within SNPs in strong linkage disequilibrium.

In order to further validate these associations a stratified analysis was carried out. Cases and controls from the two cohorts were combined together and stratified according to the geographic origin. Results are shown in Table 1.

### 3.3 The effect of the presence of non-synonymous mutations in *C3*, *CD46*, *CFH*, *CFI* and *CFB* genes

To investigate the interaction between SNPs and non-synonymous mutations in complement genes, we considered patients with and without identified mutations separately. For this analysis we focused on the Paris cohort because all the individuals within this cohort had been screened for mutations in *CFH*, *CFI*, *CD46*, *CFB* and *C3*. Table 2 summarises the mutations detected.

After application of filtering criteria (as described in Section 2.3), we analysed 467 SNPs in 75 cases with mutations and 470 SNPs within 36 cases without mutations. Both sets were compared with 140 controls from France.

Significant differences between the two subsets were observed. For SNPs within *CD46* the strength of the association with aHUS increased when the patient cohort with mutations was analysed separately. In contrast no significant association between aHUS and *CD46* SNPs was found in cases without a complement gene mutation (Table 3). An association was found for SNPs within *CFH*, *CFHR2* and *CFHR4* in both subsets.

In the Newcastle cohort (data not shown) the prevalence of mutations in aHUS patients was lower (Paris 66%, Newcastle 33%).

Consistent with the Paris cohort for all of the SNPs in *CD46*, which were associated with aHUS the *p*-values were lower (10–100 fold) in the group with mutations, suggesting that the *CD46* SNPs have a stronger effect in the presence of a known mutation. This is consistent with the data from the Paris cohort.

### 3.4 SNPs with a significant association with aHUS in one cohort

No association between aHUS and a SNP in any other complement gene was replicated in both populations. In the Newcastle cohort there were two SNPs both within the CD11b gene, which were associated with aHUS (rs9937837 and rs7499077; *p*-value < 10^−3^). In the Paris cohort there were additional SNPs both in and outside the RCA cluster that associated with aHUS but were not replicated in the Newcastle dataset. *CFHR4* contained 4 further SNPs associated with aHUS (*p*-value < 5 × 10^−3^) while *CFH* showed a further 7. Associated SNPs were also found within *CR2* (rs12032512; *p*-value = 1.74 × 10^−3^) and *C8G* (rs2071006; *p*-value < 10^−2^).

A list of 10 SNPs with the lowest *p*-values within each cohort is shown in Table 4.

### 3.5 LD estimation within *CD46* and *CFH* and haplotype analysis

Linkage disequilibrium (LD) analyses were performed with the two control groups both separately (data not shown) and combined. As the results of both analyses were similar, only the results from combined analysis are discussed.

**Fig. 3:**
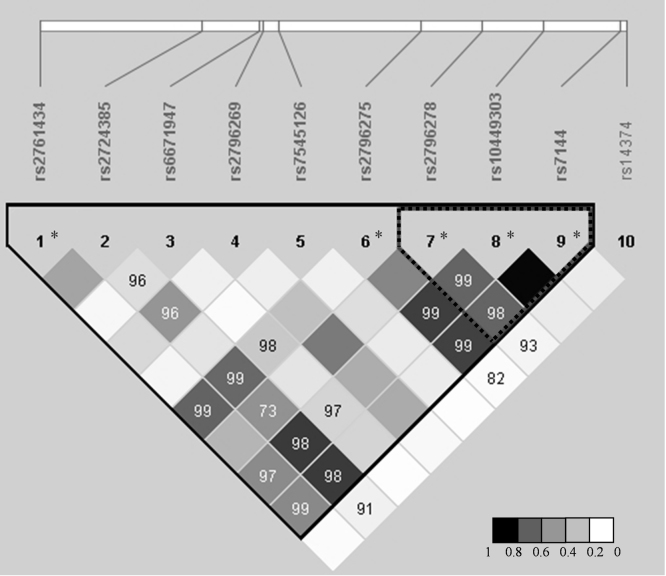
Linkage disequilibrium plot of the genotyped SNPs within *CD46*. Calculation of linkage disequilibrium parameters (*r*^2^ and *D*′) based on genotype data from 477 healthy controls was performed using Haploview. Values in each diamond are *D*′. Empty squares indicate that *D*′ is 1. Darker gray shading in the box plot indicates higher *r*^2^ according to the scale. Dotted triangle indicates a block of 12 kb with high *r*^2^ (*r*^2^ > 0.6). Black triangle shows a block of high linkage disequilibrium defined by *D*′ coefficient. *, aHUS associated SNPs.

Linkage disequilibrium between the 21 SNPs within *CFH* and 10 SNPs within *CD46* gene was estimated. The LD plot for *CD46* (Fig. 3) showed high correlation (*r*^2^ > 0.6) in a region that spans approximately 12 kb on chromosome 1. This region contains three SNPs (rs2796278, rs10449303 and rs7144) strongly associated with aHUS. If *D*′ as a linkage disequilibrium coefficient is used the LD plot shows one block spanning over 50 kb on chromosome 1 (from rs2761434 to rs7144).

**Fig. 4:**
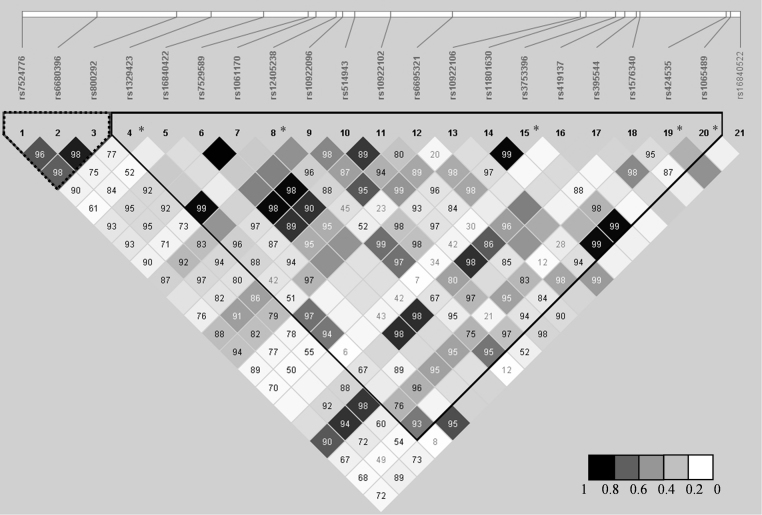
Linkage disequilibrium plot of the genotyped SNPs within CFH. Calculation of linkage disequilibrium parameters (*r*^2^ and *D*′) based on genotype data from 477 healthy controls was performed using Haploview. Values in each diamond are *D*′. Empty squares indicate that *D*′ is 1. Darker gray shading in the box plot indicates higher *r*^2^ according to the scale. Dotted triangle indicates a block of 12 kb with high *r*^2^ (*r*^2^ > 0.6). Black triangle shows a block of high linkage disequilibrium defined by *D*′ coefficient. *, aHUS associated SNPs.

The LD plot for *CFH* (Fig. 4), showed a region of 19 kb with high correlation (*r*^2^ > 0.6) containing three SNPs (rs7524776, rs6680396 and rs800292). No other region displayed a correlation coefficient > 0.6 between three or more SNPs. A reason for the low correlation coefficients may be the initial choice of the SNPs to be genotyped. If the *D*′ coefficient is used a block spanning over 63 kb can be defined (from rs1329423 to rs1065489). This block, as shown in Fig. 4, contains the 5 SNPs associated with aHUS.

Haplotype analysis of the SNPs in *CD46* and *CFH* associated with aHUS was performed using the --hap-logistic option in Plink for the two case control cohorts. Five possible haplotypes for *CD46* were observed in both cohorts. The odds ratio for developing aHUS was calculated for each haplotype. A significant difference between cases and controls were observed for two haplotypes in both cohorts. The haplotype *CD46*_ACAGC_ (Table 5) is strongly associated with the risk of the disease (odds ratio 2.4 and 2.6 in Newcastle and Paris cohorts respectively; *p*-value < 10^−6^) while the haplotype *CD46*_GTCAT_ has a protective effect (odds ratio 0.5 in both cohorts; *p*-value < 5 × 10^−4^) (Table 5). As would be predicted from the single SNP analysis the presence of the ‘at risk’ haplotype was only important in the presence of a complement gene mutation (Table 6).

A similar trend was found in cases and controls of both cohorts for the *CFH* haplotype. We identified one haplotype block *CFH*_GTGAT_ strongly associated with the risk of aHUS (odds ratio 2.2 and 2.9 in Newcastle and Paris cohorts respectively; *p*-value < 10^−4^) and one haplotype *CFH*_AGATG_ with a protective effect (odds ratio 0.5 and 0.4 in Newcastle and Paris cohorts respectively; *p*-value < 10^−3^) (Table 5).

## 4 Discussion

In this study we have examined the hypothesis that common genetic variants in complement genes apart from *CFH*, *CD46* and the *CFHRs* are associated with aHUS. In two independent cohorts of aHUS patients we have genotyped 501 SNPs in 47 complement genes. We were unable to find a replicable association in the two cohorts in any other complement gene apart from *CFH*, *CD46* and the *CFHRs*. In particular there was no replicable association with *CFI*, *C3* and *CFB* despite these being genes, like *CFH* and *CD46*, where aHUS associated mutations have been detected. In both cohorts we have confirmed that *CFH* and *CD46* alleles are significantly more frequent in aHUS patients. Caprioli et al. (2003) first reported this for *CFH* in 2003 showing that three *CFH* SNPs −331C>T (rs3753394), c.2016A>G Gln672Gln (rs3753396) and c.2808G>T Glu936Asp (rs1065489) were associated with aHUS. In 2005 we confirmed these findings in two independent cohorts of patients and also examined the allele frequency of six *CD46* SNPs (Fremeaux-Bacchi et al., 2005). One of these (*CD46* c.4070T>C, rs7144) was associated with aHUS in both cohorts. Subsequently Esparza-Gordillo et al. (2005) defined a haplotype in *CD46* (*CD46*_GGAAC_) that was associated with an increased risk of aHUS. *CD46*_GGAAC_ is defined by the following SNPs −652A>**G** (rs2796267), −366A>**G** (rs2796268), IVS9 −78G>**A** (rs1962149), IVS12 +638G>**A** (rs859705) and c.4070T>**C** (rs7144) where the at-risk alleles are in bold. Pickering et al. (2007) also subsequently defined a haplotype in *CFH* (*CFH*-H3) associated with an increased risk of aHUS. This was subsequently updated by Rodriguez de Cordoba and de Jorge (2008). *CFH*-H3 is defined by the following SNPs −331C>**T** (rs3753394), c.184**G**>A Val62Ile (rs800292), c.1204**T**>C p.Tyr402His (rs1061170), c.2016A>**G** p.Gln672Gln (rs3753396), IVS15 −543**G**>A intron 15 (rs1410996) and c.2808G>**T** p.Glu936Asp (rs1065489) where the at-risk alleles are in bold. This includes the three SNPs originally reported by Caprioli et al. (2003). In the same study Pickering et al. (2007) also defined a further *CFH* haplotype (CFH-H2) that protects against the development of aHUS. *CFH*-H2 is defined by the following SNPs −331**C**>T (rs3753394), c.184G>**A** Val62Ile (rs800292), c.1204**T**>C p.Tyr402His (rs1061170), c.2016**A**>G p.Gln672Gln (rs3753396), IVS15 −543G>**A** intron 15 (rs1410996) and c.2808**G**>T p.Glu936Asp (rs1065489) where the protective alleles are in bold.

In this study we have confirmed the presence of an at-risk *CD46* haplotype but in addition have identified a protective haplotype. It is probable that the at-risk *CD46* haplotype that we have defined in this study is the same as the previously described *CD46*_GGAAC_. However, only one of the SNPs (*CD46* c.4070T>C; rs7144) that define *CD46*_GGAAC_ also defines our at-risk haplotype. While our at-risk haplotype is, like *CD46*_GGAAC_, defined by the C allele of rs7144 there are two other rare haplotypes that are also defined by the C allele. Both *CD46* haplotypes occur in a region in strong linkage disequilibrium spanning over 59 kb of the *CD46* gene (Supplementary Fig. 1).

Like Pickering et al. (2007) we have identified both an at-risk and a protective *CFH* haplotype. Two of the *CFH* SNPs (rs3753396 and rs1065489) that define our at-risk and protective *CFH* haplotypes were also used by Pickering et al. to define *CFH*-H3 and *CFH*-H2. The alleles for both rs3753396 and rs1065489 that define the at-risk and protective *CFH* haplotypes are the same in our study and that of Pickering et al. Thus, it is again probable that these are the same haplotypes. In addition to the SNPs in *CD46* and *CFH* there were SNPs within *CFHR2* and *CFHR4* (rs9427934 and rs3795341 respectively) that demonstrated a replicable association with aHUS. This would suggest that other genes in proximity to *CD46* and *CFH* within the regulators of complement activation (RCA) cluster of genes at 1q32, particularly the *CFHRs*, are susceptibility factors for aHUS. In this study we did not examine the allele frequency of the novel *CFHR1* polymorphism described by Abarrategui-Garrido et al. (2009) where the at-risk CFHR1*B has greater sequence similarity to factor H and may thus compete with it. Neither have we examined the frequency of the well described *CFHR3/1* deletion that is associated, in homozygosity, with factor H autoantibodies (Moore et al., 2010). In contrast there were no replicable associations between SNPs outside the RCA gene cluster with aHUS. It is particularly interesting that we found no replicable association between aHUS and variants in *CFI*, *CFB* and *C3*. *CFB* and *C3* are genes in which aHUS mutations have been found but they are also all genes, which encode complement activators. We speculate that it is naturally occurring variability in complement regulators rather than activators that has the predominant effect in determining manifestation of aHUS.

When data from the two populations are combined, statistically significant associations between other SNPs and aHUS can be identified. Whether these are real associations require further replication studies with greater power.

When a subgroup analysis in patients with or without complement gene mutations was performed, SNPs within *CD46* and *CFH* behaved differently. The significance of SNPs within *CD46* increased in the group of patients with a gene mutation but significance was lost in patients without mutations. This suggests that the “at risk” SNPs (and haplotype blocks) within *CD46* are most important when they co-exist with a complement gene mutation (Esparza-Gordillo et al., 2005). In contrast SNPs within *CFH* were statistically associated with aHUS in both populations. This is at variance with our previous study (Fremeaux-Bacchi et al., 2005) which showed that the association between variations in *CFH* and *CD46* and aHUS was present irrespective of the presence of a mutation but is consistent with the findings of Esparza-Gordillo et al. (2005) who showed that the association with *CD46* was due almost exclusively to the aHUS patients with mutations in *CFH*, *CD46* and *CFI*.

In this study we have, therefore, confirmed and emphasised the pivotal importance of common genetic variants in *CFH* and *CD46* in predisposing to aHUS. That common genetic variants in complement genes can collectively also functionally determine a disease such as aHUS has recently been established. Heurich et al. (2011) have shown that non-synonymous variants encoding isoforms in C3 (p.Arg102Gly), factor B (Arg32Gln) and factor H (Val62Ile) can at a protein level combine functionally to influence susceptibility to complement driven diseases such as aHUS. This functional combination they suggest determines an individual's “complotype”. While we have not in this study established that common genetic variants in *CFB*, *CFI* and *C3* on their own are associated with aHUS we cannot exclude the possibility of an additive effect of such variants. That the functional activity of the multiple activators and regulators of the complement pathway can combine to determine susceptibility to diseases such as aHUS is a concept that is worthy of further study.

## Role of the funding source

This study was supported by grant provided by the Medical Research Council (G0701325). The funding source had no involvement in study design; in the collection, analysis, and interpretation of data; in the writing of the report; and in the decision to submit the paper for publication.

## Contributions

L.E. performed the research, collected data, performed statistical analysis and co-wrote the paper. T.H.J.G. designed the research, interpreted data and reviewed the manuscript. L.S. performed research and collected data. M.E.W. was involved in the development of the complement SNP panel and contributed analytical tools. S.H. Sacks was involved in the development of the complement SNP panel and contributed analytical tools. H.J.C. analysed and interpreted data and reviewed the manuscript. V.F.-B. interpreted data, provided vital reagents and reviewed the manuscript. N.S.S. designed the research, interpreted data and co-wrote the manuscript.

## Conflict of interest

The authors declare no competing interests.

## Figures and Tables

**Table 1:** Association between aHUS and SNPs within complement genes. Only SNPs with a *p*-value less than 10^−2^ replicated in both cohorts are shown.

SNP	Ch	Gene	A1	A2	Newcastle		Paris		Combined		
					*p*-Values	OR	*p*-Values	OR	CMH *p*-Values^d^	OR	BD *p*-Values
rs2761434^a^	1	Intergene *CR1L*-*CD46*	A	G	3.05E−05	2.4	8.53E−06	2.9	9.38E−14	2.7	0.5
rs2796275		*CD46*	C	T	1.05E−04	2.2	2.08E−04	2.3	1.51E−10	2.2	0.9
rs10449303^a^			G	A	5.70E−04	2.1	1.22E−04	2.3	1.18E−09	2.1	0.9
rs7144^b^			C	T	6.17E−04	2.1	5.13E−04	2.1	8.21E−09	2.1	0.8
rs2796278			C	A	9.25E−04	0.5	5.11E−04	0.5	1.78E−08	0.5	0.9
rs1329423		*CFH*	G	A	3.33E−03	1.9	1.25E−05	2.5	6.3E−10	2.2	0.2
rs12405238			T	G	4.28E−03	1.8	2.24E−05	2.5	2.29E−09	2.2	0.3
rs3753396^c^			G	A	1.35E−03	2.1	2.70E−05	2.7	3.50E−11	2.5	0.3
rs424535			A	T	3.16E−03	1.8	4.50E−05	0.4^e^	4.47E−09	2.1	0.3
rs1065489^c^			T	G	1.60E−03	2.1	1.19E−05	2.8	1.90E−11	2.6	0.2
rs3795341		*CFHR4*	T	C	2.13E−03	2.0	1.52E−05	2.9	1.08E−10	2.4	0.2
rs9427934		*CFHR2*	A	G	4.24E−03	1.8	5.54E−05	2.3	3.65E−08	2.0	0.4

a SNPs in strong linkage disequilibrium with SNPs already described by Fremeaux-Bacchi et al. (2005) and Esparza-Gordillo et al. (2005) as shown in Supplementary Fig. 1.

b Already reported association with aHUS by Fremeaux-Bacchi et al. (2005) and Esparza-Gordillo et al. (2005).

c Already reported association with aHUS by Fremeaux-Bacchi et al. (2005).

d In all cases, the joint *p*-value is less than 1E−04, which represents the conservative Bonferroni threshold for multiple testing across all the SNPs in this study.

e Odds ratio allele T.

**Table 2:** Mutations in complement genes from aHUS cases in the Paris cohort.

Total cases	Mutations	No mutations	*C3*	*CD46*	*CFH*	*CFI*	CFB	Combined
126	84	42	14 (17%)	14 (17%)	24 (29%)	14 (17%)	4 (5%)	14 (17%)

**Table 3:** Association analysis of aHUS patients within the Paris cohort according to whether a mutation in *CFH*, *CFI*, *CD46*, *C3* or *CFB* has been identified. Only SNPs previously associated with aHUS in the whole Paris cohort and replicated in Newcastle cohort are shown.

SNP	Ch	Gene	A1	A2	Whole Paris cohort	Cases with complement mutations	Cases without complement mutations
					*p*-Values	*p*-Values	*p*-Values
rs2761434	1	Intergene *CR1L-CD46*	A	G	8.53E−06	3.02E−07	3.09E−01
rs2796275		*CD46*	C	T	2.08E−04	1.73E−05	4.28E−01
rs10449303			G	A	1.22E−04	1.51E−05	3.29E−01
rs7144			C	T	5.13E−04	5.68E−05	4.87E−01
rs2796278			C	A	5.11E−04	3.49E−04	1.60E−01
rs1329423		*CFH*	G	A	1.25E−05	1.71E−03	2.30E−05
rs12405238			T	G	2.24E−05	2.11E−03	5.13E−05
rs3753396			G	A	2.70E−05	2.51E−04	1.42E−03
rs424535			A	T	4.50E−05	3.06E−03	3.78E−04
rs1065489			T	G	1.19E−05	8.21E−05	1.72E−03
rs9427934		*CFHR2*	A	G	1.52E−05	2.52E−03	3.13E−04
rs3795341		*CFHR4*	T	C	5.54E−05	2.37E−04	5.79E−04

**Table 4:**
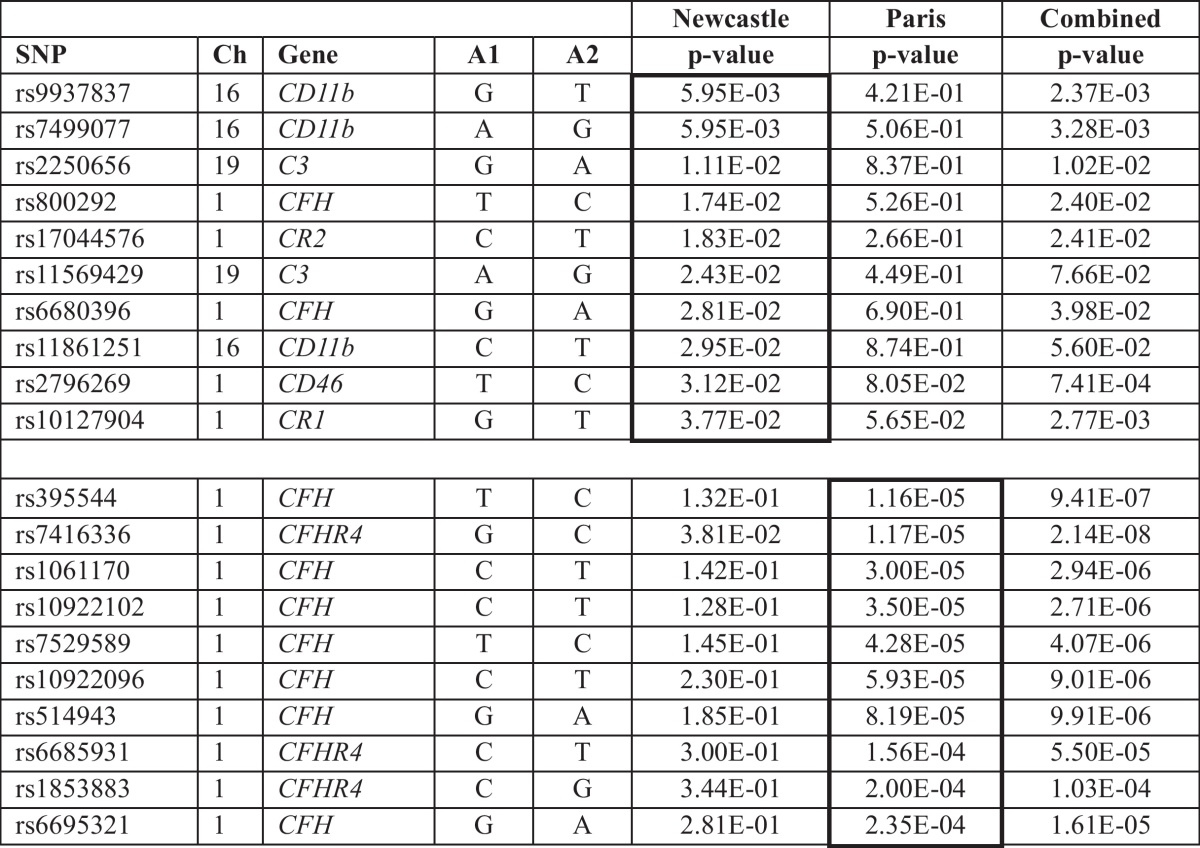
The ten lowest p-values identified in the Newcastle (upper) and Paris (lower) cohorts which were not replicated in both cohorts.

**Table 5:** Haplotype analysis of associated SNPs within *CD46* and *CFH* genes in the Newcastle and Paris cohorts. CD 46 haplotype is defined by SNPs: rs2761434 (intergenic position CR1L-CD46), rs2796275, rs2796278, rs10449303, rs7144. CFH haplotype is specified by SNPs: rs1329423, rs12405238, rs3753396, rs424535, rs1065489.

*CD46*	SNPs					Newcastle				Paris			
Haplotype	rs2761434	rs2796275	rs2796278	rs10449303	rs7144	Frequency cases	Frequency controls	OR	*p*-Value	Frequency cases	Frequency controls	OR	*p*-Value
aACAGC	A	C	A	G	C	0.43	0.23	2.4	7.34E−07	0.44	0.21	2.6	3.52E−07
GCAGC	G	C	A	G	C	0.09	0.09	0.9	8.05E−01	0.07	0.10	0.7	2.62E−01
GTAGC	G	T	A	G	C	0.05	0.07	0.7	3.16E−01	0.05	0.05	0.9	7.56E−01
GTAAT	G	T	A	A	T	0.11	0.12	0.9	7.35E−01	0.09	0.10	0.9	6.84E−01
bGTCAT	G	T	C	A	T	0.33	0.49	0.5	2.53E−04	0.36	0.54	0.5	2.60E−04

*CFH*	SNPs					Newcastle				Paris			
Haplotype	rs1329423	rs12405238	rs3753396	rs424535	rs1065489	Frequency cases	Frequency controls	OR	*p*-Value	Frequency cases	Frequency controls	OR	*p*-Value
aGTGAT	G	T	G	A	T	0.29	0.15	2.2	6.44E−05	0.40	0.17	2.9	1.28E−07
GTAAG	G	T	A	A	G	0.04	0.04	1	9.68E−01	0.07	0.08	0.9	7.66E−01
AGAAG	A	G	A	A	G	0.18	0.18	1	8.75E−01	0.17	0.17	1	9.55E−01
GTATG	G	T	A	T	G	0.06	0.04	1.3	4.88E−01	0.02	0.02	0.6	5.00E−01
bAGATG	A	G	A	T	G	0.43	0.58	0.5	7.57E−04	0.35	0.57	0.4	8.91E−07

a aHUS *CD46*_ACAGC_ and *CFH*_GTGAT_ at-risk haplotypes.

b aHUS *CD46*_GTCAT_ and *CFH*_AGATG_ protective haplotypes.

**Table 6:** *CD46* haplotype in the two subsets of aHUS patients within Paris cohort: cases with and without mutation. Only haplotypes previously associated with aHUS in the whole Paris cohort and replicated in the Newcastle cohort are shown.

Cohort	Haplotype	Frequency	OR	*p*-Value
Cases with complement mutations	aACAGC	0.31	3.3	4.54E−08
	GCAGC	0.08	0.6	2.48E−01
	GTAGC	0.05	1.0	9.92E−01
	GTAAT	0.09	0.6	1.75E−01
	bGTCAT	0.46	0.5	1.47E−04

Cases without complement mutations	aACAGC	0.22	1.5	1.89E−01
	GCAGC	0.09	0.9	8.06E−01
	GTAGC	0.05	1.2	7.69E−01
	GTAAT	0.11	1.5	2.61E−01
	bGTCAT	0.51	0.7	1.35E−01

a aHUS *CD46*_ACAGC_ at-risk haplotypes.

b aHUS *CD46*_GTCAT_ protective haplotype.
