# Supplementary material for: Common genetic variants in complement genes other than CFH, CD46 and the CFHRs are not associated with aHUS
Source: Mol Immunol. 2012 Jan;49(4):640–8. doi: 10.1016/j.molimm.2011.11.003 (PMC3438446; doi:10.1016/j.molimm.2011.11.003)
Supplement: Supplementary file 1 [file mmc1.zip › mmc1.docx]

**Supplementary figure 1: Linkage disequilibrium plot of the genotyped SNPs within *CD46***. Calculation of linkage disequilibrium D’ coefficient is based on genotype data from 477 healthy controls and was performed using Haploview. Values in each diamond are D’. Empty squares indicate that D’ is 1. Position of the SNPs already associated with aHUS from Fremeaux-Bacchi (2005) and Esparza-Gordillo (2005) are shown. SNPs genotyped by both Fremeaux-Bacchi(2005) and Esparza-Gordillo (2005) (rs2796267, rs2796268, rs1962149) are shown in blue. SNPs genotyped by Fremeaux-Bacchi (2005) (rs2724374) or Esparza-Gordillo (2005) (rs859705) are shown in red. SNP rs7144 genotyped by Fremeaux-Bacchi^23^, Esparza-Gordillo (2005) and this manuscript is shown in bold. The *CD46* haplotype *CD46*_GGAAC_ inferred by Esparza-Gordillo (2005) (black) and haplotype *CD46*_ACAGC_ inferred in this manuscript (red) are displayed.

**
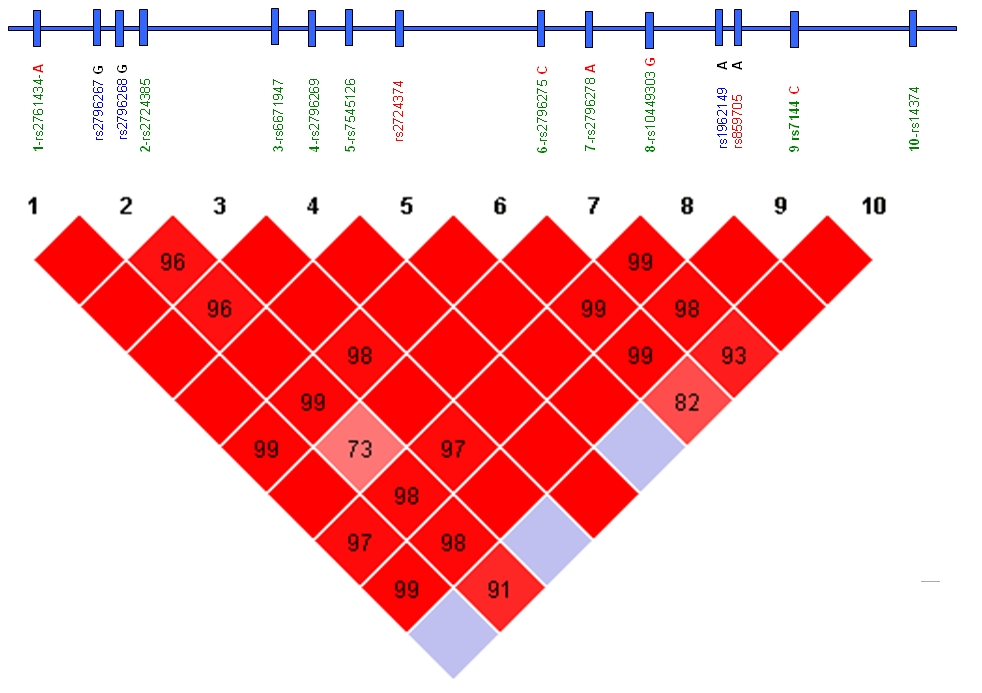
**

**Supplementary table 1**: Selected 501 SNPs analysed in this study

| **SNP** | **Function** | **Chromosome** | **Genetic distance (bp)** | **Gene ID** |
| --- | --- | --- | --- | --- |
| rs1474891 | intron | 1 | 11004467 | *TARDBP* |
| rs12711521 | nonsynonymous | 1 | 11013503 | *MASP2* |
| rs12136082 | intron | 1 | 11024862 | *MASP2* |
| rs292001 | intron | 1 | 22837545 | *C1QA* |
| rs12740591 | intron | 1 | 22837590 | *C1QA* |
| rs12073436 | intron | 1 | 22843495 | *C1QC* |
| rs294185 | intron | 1 | 22844479 | *C1QC* |
| rs291990 | near gene-5 | 1 | 22852242 | *C1QB* |
| rs12756603 | intron | 1 | 22854463 | *C1QB* |
| rs629409 | intron | 1 | 22859325 | *C1QB* |
| rs292010 | NA | 1 | 22862332 | intergenic |
| rs10794501 | intron | 1 | 27572036 | *FCN3* |
| rs662377 | intron | 1 | 57097100 | *C8A* |
| rs2405237 | intron | 1 | 57113134 | *C8A* |
| rs652785 | nonsynonymous | 1 | 57113315 | *C8A* |
| rs2300955 | intron | 1 | 57116750 | *C8A* |
| rs6694643 | intron | 1 | 57121995 | *C8A* |
| rs6697228 | intron | 1 | 57122052 | *C8A* |
| rs6683663 | intron | 1 | 57129064 | *C8A* |
| rs6686359 | intron | 1 | 57129168 | *C8A* |
| rs1418471 | intron | 1 | 57131889 | *C8A* |
| rs6696924 | intron | 1 | 57132516 | *C8A* |
| rs6699859 | intron | 1 | 57133038 | *C8A* |
| rs11206932 | intron | 1 | 57134595 | *C8A* |
| rs679350 | intron | 1 | 57137400 | *C8A* |
| rs12116668 | intron | 1 | 57143721 | *C8A* |
| rs619545 | intron | 1 | 57145179 | *C8A* |
| rs624298 | intron | 1 | 57147122 | *C8A* |
| rs1774900 | intron | 1 | 57149737 | *C8A* |
| rs638919 | intron | 1 | 57149760 | *C8A* |
| rs17300936 | nonsynonymous | 1 | 57155946 | *C8A* |
| rs17301146 | intron | 1 | 57172849 | *C8B* |
| rs17301153 | intron | 1 | 57173572 | *C8B* |
| rs594661 | intron | 1 | 57174570 | *C8B* |
| rs669358 | intron | 1 | 57177327 | *C8B* |
| rs6669836 | intron | 1 | 57178980 | *C8B* |
| rs656598 | intron | 1 | 57182733 | *C8B* |
| rs12085435 | nonsynonymous | 1 | 57187898 | *C8B* |
| rs658285 | intron | 1 | 57188923 | *C8B* |
| rs647571 | intron | 1 | 57192195 | *C8B* |
| rs1013579 | nonsynonymous | 1 | 57195072 | *C8B* |
| rs12067507 | nonsynonymous | 1 | 57195099 | *C8B* |
| rs683916 | intron | 1 | 57198062 | *C8B* |
| rs599857 | intron | 1 | 57201177 | *C8B* |
| rs2236217 | intron | 1 | 57203393 | *C8B* |
| rs4915309 | NA | 1 | 170174251 | intergenic |
| rs7524776 | intron | 1 | 194889960 | *CFH* |
| rs6680396 | intron | 1 | 194899093 | *CFH* |
| rs800292 | nonsynonymous | 1 | 194908856 | *CFH* |
| rs1329423 | intron | 1 | 194913010 | *CFH* |
| rs16840422 | intron | 1 | 194919457 | *CFH* |
| rs7529589 | intron | 1 | 194924902 | *CFH* |
| rs1061170 | nonsynonymous | 1 | 194925860 | *CFH* |
| rs12405238 | intron | 1 | 194928236 | *CFH* |
| rs10922096 | intron | 1 | 194929082 | *CFH* |
| rs514943 | intron | 1 | 194930536 | *CFH* |
| rs10922102 | intron | 1 | 194934910 | *CFH* |
| rs6695321 | intron | 1 | 194942484 | *CFH* |
| rs10922106 | intron | 1 | 194958087 | *CFH* |
| rs11801630 | intron | 1 | 194958771 | *CFH* |
| rs3753396 | synonymous | 1 | 194962365 | *CFH* |
| rs419137 | intron | 1 | 194963498 | *CFH* |
| rs395544 | intron | 1 | 194964895 | *CFH* |
| rs1576340 | intron | 1 | 194965334 | *CFH* |
| rs424535 | intron | 1 | 194975846 | *CFH* |
| rs1065489 | nonsynonymous | 1 | 194976397 | *CFH* |
| rs16840522 | intron | 1 | 194977539 | *CFH* |
| rs411854 | intron | 1 | 195028740 | *CFHR3* |
| rs16840561 | nonsynonymous | 1 | 195067646 | *CFHR1* |
| rs10754206 | NA | 1 | 195087547 | intergenic CFHR1-CFHR4 |
| rs7519492 | NA | 1 | 195106748 | intergenic CFHR1-CFHR4 |
| rs6685931 | Intron | 1 | 195133856 | *CFHR4* |
| rs7416336 | Intron | 1 | 195138798 | *CFHR4* |
| rs1853883 | Intron | 1 | 195148223 | *CFHR4* |
| rs4915559 | Intron | 1 | 195153393 | *CFHR4* |
| rs1971579 | Intron | 1 | 195153804 | *CFHR4* |
| rs3795341 | intron | 1 | 195153897 | *CFHR4* |
| rs10494745 | nonsynonymous | 1 | 195154080 | *CFHR4* |
| rs1853881 | intron | 1 | 195182368 | *CFHR2* |
| rs6674522 | intron | 1 | 195182982 | *CFHR2* |
| rs9427934 | intron | 1 | 195189483 | *CFHR2* |
| rs3748557 | intron | 1 | 195213492 | *CFHR5* |
| rs12116643 | intron | 1 | 195239806 | *CFHR5* |
| rs10922153 | UTR-3 | 1 | 195245238 | *CFHR5* |
| rs7555070 | NA | 1 | 195248777 | intergenic CFHR5-F13B |
| rs4403634 | intron | 1 | 205334714 | *C4BPB* |
| rs8942 | synonymous | 1 | 205336542 | *C4BPB* |
| rs11120218 | intron | 1 | 205345074 | *C4BPA* |
| rs17186575 | intron | 1 | 205351774 | *C4BPA* |
| rs17186582 | intron | 1 | 205352254 | *C4BPA* |
| rs17020983 | intron | 1 | 205354920 | *C4BPA* |
| rs2842704 | intron | 1 | 205356059 | *C4BPA* |
| rs4425986 | intron | 1 | 205360403 | *C4BPA* |
| rs17021100 | intron | 1 | 205360630 | *C4BPA* |
| rs7545083 | intron | 1 | 205362511 | *C4BPA* |
| rs2842707 | intron | 1 | 205364107 | *C4BPA* |
| rs1126618 | synonymous | 1 | 205364303 | *C4BPA* |
| rs2491393 | intron | 1 | 205366882 | *C4BPA* |
| rs4844573 | nonsynonymous | 1 | 205371523 | *C4BPA* |
| rs4571969 | intron | 1 | 205372211 | *C4BPA* |
| rs12087872 | intron | 1 | 205373156 | *C4BPA* |
| rs12043615 | intron | 1 | 205381011 | *C4BPA* |
| rs2039544 | NA | 1 | 205486540 | intergenic |
| rs11584921 | NA | 1 | 205508598 | intergenic |
| rs1652333 | intergenic | 1 | 205537083 | intergenic LOC100129101-CR1 |
| rs7533852 | intron | 1 | 205562050 | *CD55* |
| rs7527218 | NA | 1 | 205690452 | intergenic |
| rs3813946 | UTR-5 | 1 | 205694316 | *CR2* |
| rs12139795 | intron | 1 | 205697828 | *CR2* |
| rs17044525 | intron | 1 | 205699419 | *CR2* |
| rs17044576 | intron | 1 | 205701293 | *CR2* |
| rs1048971 | synonymous | 1 | 205712945 | *CR2* |
| rs17615 | nonsynonymous | 1 | 205713085 | *CR2* |
| rs4317805 | intron | 1 | 205719615 | *CR2* |
| rs12032512 | intron | 1 | 205719801 | *CR2* |
| rs17258982 | nonsynonymous | 1 | 205719987 | *CR2* |
| rs6540433 | nonsynonymous | 1 | 205720018 | *CR2* |
| rs17186771 | intron | 1 | 205721816 | *CR2* |
| rs1041250 | intron | 1 | 205722438 | *CR2* |
| rs6690215 | intron | 1 | 205722673 | *CR2* |
| rs6665881 | intron | 1 | 205723228 | *CR2* |
| rs12021671 | intron | 1 | 205724768 | *CR2* |
| rs2182911 | intron | 1 | 205726694 | *CR2* |
| rs2182913 | intron | 1 | 205727001 | *CR2* |
| rs7519408 | intron | 1 | 205727912 | *CR2* |
| rs9429944 | intron | 1 | 205736930 | *CR1* |
| rs1831150 | intron | 1 | 205737061 | *CR1* |
| rs1571344 | intron | 1 | 205737551 | *CR1* |
| rs1887632 | intron | 1 | 205738244 | *CR1* |
| rs2025935 | intron | 1 | 205742278 | *CR1* |
| rs9429945 | intron | 1 | 205743391 | *CR1* |
| rs11117949 | intron | 1 | 205748879 | *CR1* |
| rs6656401 | intron | 1 | 205758672 | *CR1* |
| rs3886100 | intron | 1 | 205805750 | *CR1* |
| rs12038575 | intron | 1 | 205806895 | *CR1* |
| rs10127904 | intron | 1 | 205814730 | *CR1* |
| rs614709 | intron | 1 | 205819898 | *CR1* |
| rs2274567 | nonsynonymous | 1 | 205820244 | *CR1* |
| rs3737002 | nonsynonymous | 1 | 205827396 | *CR1* |
| rs17259045 | nonsynonymous | 1 | 205849330 | *CR1* |
| rs6691117 | nonsynonymous | 1 | 205849554 | *CR1* |
| rs12734030 | intron | 1 | 205860587 | *CR1* |
| rs2296160 | nonsynonymous | 1 | 205861943 | *CR1* |
| rs12034383 | intron | 1 | 205870218 | *CR1* |
| rs6661764 | intron | 1 | 205875008 | *CR1* |
| rs2761434 | intergenic CR1L-CD46 | 1 | 205984122 | intergenic CR1L-CD46 |
| rs12062235 | NA | 1 | 205988416 | *CD46* |
| rs2724385 | intron | 1 | 205998151 | *CD46* |
| rs6671947 | intron | 1 | 206003114 | *CD46* |
| rs2796269 | intron | 1 | 206003509 | *CD46* |
| rs7545126 | intron | 1 | 206004859 | *CD46* |
| rs2796275 | intron | 1 | 206017113 | *CD46* |
| rs2796278 | intron | 1 | 206022446 | *CD46* |
| rs10449303 | intron | 1 | 206027681 | *CD46* |
| rs7144 | UTR-3 | 1 | 206034342 | *CD46* |
| rs14374 | UTR-3 | 1 | 206034880 | *CD46* |
| rs6772329 | near gene-5 | 3 | 188397297 | *RTP1* |
| rs3843010 | UTR-3 | 3 | 188419741 | *MASP1* |
| rs3821801 | UTR-3 | 3 | 188419915 | *MASP1* |
| rs17040 | UTR-3 | 3 | 188420262 | *MASP1* |
| rs3733003 | intron | 3 | 188421885 | *MASP1* |
| rs3914010 | intron | 3 | 188422267 | *MASP1* |
| rs9880761 | intron | 3 | 188423991 | *MASP1* |
| rs11720718 | intron | 3 | 188424123 | *MASP1* |
| rs6762462 | intron | 3 | 188424749 | *MASP1* |
| rs1001073 | intron | 3 | 188427399 | *MASP1* |
| rs4315676 | intron | 3 | 188429116 | *MASP1* |
| rs16861755 | intron | 3 | 188429922 | *MASP1* |
| rs850317 | intron | 3 | 188430495 | *MASP1* |
| rs850316 | intron | 3 | 188430846 | *MASP1* |
| rs11923275 | intron | 3 | 188431367 | *MASP1* |
| rs10513805 | intron | 3 | 188433091 | *MASP1* |
| rs2002665 | intron | 3 | 188434166 | *MASP1* |
| rs876650 | intron | 3 | 188434334 | *MASP1* |
| rs12489890 | intron | 3 | 188435109 | *MASP1* |
| rs874603 | intron | 3 | 188435731 | *MASP1* |
| rs850313 | intron | 3 | 188435920 | *MASP1* |
| rs850312 | synonymous | 3 | 188436502 | *MASP1* |
| rs3774268 | synonymous | 3 | 188437018 | *MASP1* |
| rs696405 | intron | 3 | 188438917 | *MASP1* |
| rs12107082 | intron | 3 | 188439068 | *MASP1* |
| rs3815623 | intron | 3 | 188441736 | *MASP1* |
| rs710459 | intron | 3 | 188443176 | *MASP1* |
| rs710462 | intron | 3 | 188445824 | *MASP1* |
| rs698090 | intron | 3 | 188446994 | *MASP1* |
| rs3774275 | intron | 3 | 188447962 | *MASP1* |
| rs698092 | intron | 3 | 188452328 | *MASP1* |
| rs698093 | intron | 3 | 188452845 | *MASP1* |
| rs3733006 | intron | 3 | 188453391 | *MASP1* |
| rs698095 | intron | 3 | 188458714 | *MASP1* |
| rs1357134 | intron | 3 | 188462462 | *MASP1* |
| rs823940 | intron | 3 | 188462698 | *MASP1* |
| rs3774279 | intron | 3 | 188463795 | *MASP1* |
| rs850307 | intron | 3 | 188464162 | *MASP1* |
| rs3774282 | intron | 3 | 188464902 | *MASP1* |
| rs17375194 | intron | 3 | 188470176 | *MASP1* |
| rs12106739 | intron | 3 | 188473505 | *MASP1* |
| rs710473 | intron | 3 | 188475684 | *MASP1* |
| rs3864098 | intron | 3 | 188478554 | *MASP1* |
| rs3107217 | intron | 3 | 188479513 | *MASP1* |
| rs1717652 | intron | 3 | 188479833 | *MASP1* |
| rs698085 | intron | 3 | 188480117 | *MASP1* |
| rs698084 | intron | 3 | 188480342 | *MASP1* |
| rs3864099 | intron | 3 | 188481632 | *MASP1* |
| rs698104 | intron | 3 | 188485211 | *MASP1* |
| rs13064994 | intron | 3 | 188488710 | *MASP1* |
| rs16861893 | intron | 3 | 188491870 | *MASP1* |
| rs13089330 | near gene-5 | 3 | 188492964 | *MASP1* |
| rs17040805 | intron | 4 | 110884246 | *CFI* |
| rs6848178 | intron | 4 | 110885620 | *CFI* |
| rs6822976 | intron | 4 | 110885741 | *CFI* |
| rs9998151 | intron | 4 | 110886794 | *CFI* |
| rs6836770 | intron | 4 | 110887777 | *CFI* |
| rs7438961 | intron | 4 | 110888100 | *CFI* |
| rs2298749 | synonymous | 4 | 110900954 | *CFI* |
| rs4541508 | intron | 4 | 110901936 | *CFI* |
| rs4469075 | intron | 4 | 110910035 | *CFI* |
| rs885448 | intron | 4 | 110910108 | *CFI* |
| rs11721403 | intron | 4 | 110913018 | *CFI* |
| rs1000954 | intron | 4 | 110929483 | *CFI* |
| rs4698788 | intron | 4 | 110938983 | *CFI* |
| rs7671905 | intron | 4 | 110942335 | *CFI* |
| rs17468519 | intron | 5 | 39339216 | *C9* |
| rs155377 | intron | 5 | 39344808 | *C9* |
| rs11959122 | intron | 5 | 39346359 | *C9* |
| rs263275 | intron | 5 | 39350934 | *C9* |
| rs187875 | intron | 5 | 39351502 | *C9* |
| rs261753 | intron | 5 | 39361099 | *C9* |
| rs11953839 | intron | 5 | 39365195 | *C9* |
| rs17384711 | intron | 5 | 39365431 | *C9* |
| rs696760 | intron | 5 | 39369251 | *C9* |
| rs534663 | intron | 5 | 39376318 | *C9* |
| rs696764 | intron | 5 | 39378250 | *C9* |
| rs835209 | intron | 5 | 39386120 | *C9* |
| rs11740241 | intron | 5 | 39387223 | *C9* |
| rs3776526 | intron | 5 | 39389479 | *C9* |
| rs1421094 | intron | 5 | 39391348 | *C9* |
| rs10941432 | intron | 5 | 39395033 | *C9* |
| rs700233 | nonsynonymous | 5 | 39400311 | *C9* |
| rs13354307 | NA | 5 | 40905076 | intergenic |
| rs12153063 | near gene-5 | 5 | 40943951 | *C7* |
| rs1910016 | intron | 5 | 40947349 | *C7* |
| rs16870514 | intron | 5 | 40950922 | *C7* |
| rs324062 | intron | 5 | 40956290 | *C7* |
| rs2443040 | intron | 5 | 40964331 | *C7* |
| rs2455308 | intron | 5 | 40965270 | *C7* |
| rs12658133 | intron | 5 | 40968376 | *C7* |
| rs3792640 | intron | 5 | 40968595 | *C7* |
| rs3792642 | intron | 5 | 40968910 | *C7* |
| rs324056 | intron | 5 | 40968997 | *C7* |
| rs6875226 | intron | 5 | 40979199 | *C7* |
| rs2455313 | intron | 5 | 40982362 | *C7* |
| rs2084624 | intron | 5 | 40986565 | *C7* |
| rs1063499 | nonsynonymous | 5 | 40991318 | *C7* |
| rs2675982 | intron | 5 | 40996035 | *C7* |
| rs324058 | intron | 5 | 40997651 | *C7* |
| rs7713409 | intron | 5 | 41000352 | *C7* |
| rs7713884 | intron | 5 | 41000573 | *C7* |
| rs13157656 | nonsynonymous | 5 | 41000609 | *C7* |
| rs3805226 | intron | 5 | 41009762 | *C7* |
| rs3828511 | intron | 5 | 41012092 | *C7* |
| rs1078375 | intron | 5 | 41013517 | *C7* |
| rs2876849 | intron | 5 | 41015547 | *C7* |
| rs3749519 | intron | 5 | 41017132 | *C7* |
| rs1061443 | UTR-3 | 5 | 41018379 | *C7* |
| rs7732104 | near gene-3 | 5 | 41019193 | *C7* |
| rs1833863 | NA | 5 | 41125622 | intergenic |
| rs1423394 | NA | 5 | 41152600 | intergenic |
| rs1423396 | NA | 5 | 41158467 | intergenic |
| rs9200 | UTR-3 | 5 | 41178363 | *C6* |
| rs918608 | intron | 5 | 41188446 | *C6* |
| rs3805713 | intron | 5 | 41189118 | *C6* |
| rs2301247 | intron | 5 | 41190285 | *C6* |
| rs10512764 | intron | 5 | 41193097 | *C6* |
| rs3805717 | intron | 5 | 41197278 | *C6* |
| rs1035497 | intron | 5 | 41205972 | *C6* |
| rs6883180 | intron | 5 | 41216128 | *C6* |
| rs17201075 | intron | 5 | 41221364 | *C6* |
| rs17201103 | intron | 5 | 41221403 | *C6* |
| rs6860770 | intron | 5 | 41221669 | *C6* |
| rs13177483 | intron | 5 | 41222631 | *C6* |
| rs4400166 | intron | 5 | 41224048 | *C6* |
| rs11951782 | intron | 5 | 41227310 | *C6* |
| rs10473238 | intron | 5 | 41231987 | *C6* |
| rs11957341 | intron | 5 | 41233229 | *C6* |
| rs1801033 | nonsynonymous | 5 | 41235716 | *C6* |
| rs1444903 | intron | 5 | 41240060 | *C6* |
| rs10512766 | intron | 5 | 41249092 | *C6* |
| rs16871040 | intron | 5 | 41269943 | *C6* |
| rs7746553 | intron | 6 | 32003952 | *C2* |
| rs9332739 | nonsynonymous | 6 | 32011783 | *C2* |
| rs550605 | intron | 6 | 32015126 | *C2* |
| rs1265905 | intron | 6 | 32017920 | *C2* |
| rs547154 | intron | 6 | 32018917 | *C2* |
| rs9332730 | intron | 6 | 32019988 | *CFB/C2* |
| rs4151667 | nonsynonymous | 6 | 32022003 | *CFB* |
| rs12614 | nonsynonymous | 6 | 32022158 | *CFB* |
| rs641153 | nonsynonymous | 6 | 32022159 | *CFB* |
| rs1048709 | synonymous | 6 | 32022914 | *CFB* |
| rs537160 | intron | 6 | 32024379 | *CFB* |
| rs541862 | intron | 6 | 32024930 | *CFB* |
| rs4151657 | intron | 6 | 32025519 | *CFB* |
| rs1270942 | intron | 6 | 32026839 | *CFB* |
| rs4151671 | intron | 6 | 32026882 | *CFB* |
| rs2072633 | intron | 6 | 32027557 | *CFB* |
| rs17057444 | NA | 8 | 27509710 | *CLU* |
| rs3087554 | UTR-3 | 8 | 27511359 | *CLU* |
| rs2279590 | intron | 8 | 27512170 | *CLU* |
| rs9331930 | intron | 8 | 27514211 | *CLU* |
| rs11136000 | intron | 8 | 27520436 | *CLU* |
| rs867231 | intron | 8 | 27523309 | *CLU* |
| rs867230 | intron | 8 | 27524420 | *CLU* |
| rs9331888 | intron | 8 | 27524779 | *CLU* |
| rs2300932 | intron | 9 | 22810257 | *C5* |
| rs2416815 | NA | 9 | 94472478 | intergenic |
| rs2191959 | intron | 9 | 122723655 | *TRAF1* |
| rs17612 | nonsynonymous | 9 | 122765747 | *C5* |
| rs7026551 | intron | 9 | 122772954 | *C5* |
| rs2269066 | intron | 9 | 122776839 | *C5* |
| rs7037673 | intron | 9 | 122780305 | *C5* |
| rs4837805 | intron | 9 | 122786076 | *C5* |
| rs10818494 | intron | 9 | 122786259 | *C5* |
| rs16910255 | intron | 9 | 122796804 | *C5* |
| rs10818495 | intron | 9 | 122797008 | *C5* |
| rs7040033 | intron | 9 | 122798865 | *C5* |
| rs12685344 | intron | 9 | 122804812 | *C5* |
| rs17611 | nonsynonymous | 9 | 122809021 | *C5* |
| rs7027797 | intron | 9 | 122811620 | *C5* |
| rs25681 | synonymous | 9 | 122819826 | *C5* |
| rs25682 | synonymous | 9 | 122819925 | *C5* |
| rs992670 | intron | 9 | 122821591 | *C5* |
| rs2230214 | synonymous | 9 | 122822120 | *C5* |
| rs2416810 | intron | 9 | 122825021 | *C5* |
| rs17220750 | intron | 9 | 122827820 | *C5* |
| rs17216529 | nonsynonymous | 9 | 122840039 | *C5* |
| rs16910280 | intron | 9 | 122844597 | *C5* |
| rs7040603 | intron | 9 | 122848041 | *C5* |
| rs1468673 | intron | 9 | 122849711 | *C5* |
| rs17514136 | UTR-5 | 9 | 136912485 | *FCN2* |
| rs3128627 | intron | 9 | 136912613 | *FCN2* |
| rs3128626 | intron | 9 | 136913377 | *FCN2* |
| rs4370609 | intron | 9 | 136914384 | *FCN2* |
| rs7024491 | intron | 9 | 136914905 | *FCN2* |
| rs7041446 | intron | 9 | 136915875 | *FCN2* |
| rs3128623 | intron | 9 | 136915930 | *FCN2* |
| rs12685659 | intron | 9 | 136916540 | *FCN2* |
| rs7872508 | intron | 9 | 136918708 | *FCN2* |
| rs17549193 | nonsynonymous | 9 | 136918847 | *FCN2* |
| rs7851696 | nonsynonymous | 9 | 136918912 | *FCN2* |
| rs4521835 | UTR-5 | 9 | 136919127 | *FCN2* |
| rs2989735 | near gene-3 | 9 | 136941237 | *FCN1* |
| rs1105323 | intron | 9 | 136942234 | *FCN1* |
| rs2274845 | synonymous | 9 | 136944181 | *FCN1* |
| rs2989725 | intron | 9 | 136947826 | *FCN1* |
| rs12377780 | intron | 9 | 136948782 | *FCN1* |
| rs2989726 | intron | 9 | 136949195 | *FCN1* |
| rs2071006 | synonymous | 9 | 138959725 | *C8G* |
| rs9663421 | intron | 10 | 6095610 | *IL2RA* |
| rs12722596 | intron | 10 | 6096300 | *IL2RA* |
| rs2386841 | intron | 10 | 6097738 | *IL2RA* |
| rs7899538 | intron | 10 | 6099904 | *IL2RA* |
| rs12722588 | intron | 10 | 6100439 | *IL2RA* |
| rs2274037 | intron | 10 | 6102114 | *IL2RA* |
| rs2076846 | intron | 10 | 6103259 | *IL2RA* |
| rs7093069 | intron | 10 | 6103325 | *IL2RA* |
| rs11596355 | intron | 10 | 6104187 | *IL2RA* |
| rs7900744 | intron | 10 | 6105617 | *IL2RA* |
| rs2025345 | intron | 10 | 6107694 | *IL2RA* |
| rs2228150 | synonymous | 10 | 6107975 | *IL2RA* |
| rs12722563 | intron | 10 | 6109567 | *IL2RA* |
| rs7910961 | intron | 10 | 6117802 | *IL2RA* |
| rs11256433 | intron | 10 | 6117851 | *IL2RA* |
| rs6602391 | intron | 10 | 6118038 | *IL2RA* |
| rs6602392 | intron | 10 | 6118085 | *IL2RA* |
| rs11256448 | intron | 10 | 6119485 | *IL2RA* |
| rs7072398 | intron | 10 | 6119852 | *IL2RA* |
| rs11256456 | intron | 10 | 6120718 | *IL2RA* |
| rs6602398 | intron | 10 | 6122959 | *IL2RA* |
| rs11598648 | intron | 10 | 6124031 | *IL2RA* |
| rs4749926 | intron | 10 | 6125318 | *IL2RA* |
| rs942201 | intron | 10 | 6126298 | *IL2RA* |
| rs706781 | intron | 10 | 6126391 | *IL2RA* |
| rs706780 | intron | 10 | 6127032 | *IL2RA* |
| rs11256497 | intron | 10 | 6127800 | *IL2RA* |
| rs791587 | intron | 10 | 6128705 | *IL2RA* |
| rs791589 | intron | 10 | 6129577 | *IL2RA* |
| rs791590 | intron | 10 | 6130328 | *IL2RA* |
| rs10905668 | intron | 10 | 6132061 | *IL2RA* |
| rs2476491 | intron | 10 | 6135416 | *IL2RA* |
| rs2256774 | intron | 10 | 6137171 | *IL2RA* |
| rs706779 | intron | 10 | 6138830 | *IL2RA* |
| rs706778 | intron | 10 | 6138955 | *IL2RA* |
| rs2104286 | intron | 10 | 6139051 | *IL2RA* |
| rs3118470 | intron | 10 | 6141719 | *IL2RA* |
| rs12722486 | intron | 10 | 6143768 | *IL2RA* |
| rs4935346 | NA | 10 | 54174739 | intergenic |
| rs1877134 | NA | 10 | 54184769 | intergenic |
| rs11595876 | UTR-3 | 10 | 54196768 | *MBL2* |
| rs930507 | synonymous | 10 | 54198272 | *MBL2* |
| rs1838065 | intron | 10 | 54199263 | *MBL2* |
| rs1838066 | intron | 10 | 54199331 | *MBL2* |
| rs4935047 | intron | 10 | 54200073 | *MBL2* |
| rs1800450 | nonsynonymous | 10 | 54201241 | *MBL2* |
| rs10824794 | near gene-5 | 10 | 54203195 | *MBL2* |
| rs704697 | UTR-3 | 11 | 33681356 | *CD59* |
| rs1546727 | UTR-3 | 11 | 33682798 | *CD59* |
| rs7046 | UTR-3 | 11 | 33683556 | *CD59* |
| rs11585 | UTR-3 | 11 | 33684281 | *CD59* |
| rs11032349 | UTR-3 | 11 | 33686248 | *CD59* |
| rs17760306 | intron | 11 | 33694612 | *CD59* |
| rs831636 | intron | 11 | 33696174 | *CD59* |
| rs17687664 | intron | 11 | 33696988 | *CD59* |
| rs831631 | intron | 11 | 33698141 | *CD59* |
| rs831628 | near gene-5 | 11 | 33701421 | *CD59* |
| rs831627 | near gene-5 | 11 | 33702623 | *CD59* |
| rs831626 | near gene-5 | 11 | 33702926 | *CD59* |
| rs1738548 | intron | 11 | 33708979 | *CD59* |
| rs10768024 | intron | 11 | 33711053 | *CD59* |
| rs3181274 | intron | 11 | 33711865 | *CD59* |
| rs3181268 | intron | 11 | 33713749 | *CD59* |
| rs2273121 | intron | 11 | 33714346 | *CD59* |
| rs1005511 | intron | 11 | 57123232 | *SERPING1* |
| rs2511989 | intron | 11 | 57134901 | *SERPING1* |
| rs4926 | nonsynonymous | 11 | 57138565 | *SERPING1* |
| rs11064497 | intron | 12 | 7039922 | *C1S* |
| rs11064498 | intron | 12 | 7041768 | *C1S* |
| rs7968584 | intron | 12 | 7042502 | *C1S* |
| rs2192137 | intron | 12 | 7045031 | *C1S* |
| rs1143664 | synonymous | 12 | 7045308 | *C1S* |
| rs7183 | UTR-3 | 12 | 7048280 | *C1S* |
| rs3813729 | nonsynonymous | 12 | 7133345 | *CIR* |
| rs1801046 | NA | 12 | 7133440 | *C1R* |
| rs7135975 | intron | 12 | 7134709 | *CIR* |
| rs7308467 | intron | 12 | 7134924 | *CIR* |
| rs7842 | UTR-3 | 12 | 8102454 | *C3AR1* |
| rs7954916 | intron | 12 | 8109659 | *C3AR1* |
| rs1143679 | nonsynonymous | 16 | 31184312 | *CD11B* |
| rs8048583 | intron | 16 | 31187037 | *CD11B* |
| rs7184677 | intron | 16 | 31196567 | *CD11B* |
| rs11861251 | nonsynonymous | 16 | 31196897 | *CD11B* |
| rs9937837 | intron | 16 | 31206440 | *CD11B* |
| rs9888879 | intron | 16 | 31217873 | *CD11B* |
| rs11645653 | intron | 16 | 31220356 | *CD11B* |
| rs7499077 | intron | 16 | 31225006 | *CD11B* |
| rs6565228 | intron | 16 | 31236781 | *CD11B* |
| rs11150610 | intron | 16 | 31241737 | *CD11B* |
| rs1143683 | nonsynonymous | 16 | 31244389 | *CD11B* |
| rs4077810 | intron | 16 | 31248410 | *CD11B* |
| rs7193268 | intron | 16 | 31248498 | *CD11B* |
| rs1143678 | nonsynonymous | 16 | 31250506 | *CD11B* |
| rs4597342 | UTR-3 | 16 | 31251270 | *CD11B* |
| rs4522435 | NA | 16 | 31269424 | intergenic |
| rs1629038 | intron | 19 | 811852 | *CFD* |
| rs3826945 | intron | 19 | 813912 | *CFD* |
| rs17030 | synonymous | 19 | 6628989 | *C3* |
| rs344555 | intron | 19 | 6630360 | *C3* |
| rs2277984 | intron | 19 | 6630511 | *C3* |
| rs7951 | synonymous | 19 | 6632991 | *C3* |
| rs344550 | intron | 19 | 6633953 | *C3* |
| rs11569547 | intron | 19 | 6634622 | *C3* |
| rs11569546 | intron | 19 | 6634749 | *C3* |
| rs11569545 | intron | 19 | 6634754 | *C3* |
| rs344549 | intron | 19 | 6634846 | *C3* |
| rs1389623 | intron | 19 | 6635197 | *C3* |
| rs2241394 | intron | 19 | 6636230 | *C3* |
| rs2241393 | intron | 19 | 6636304 | *C3* |
| rs11569538 | intron | 19 | 6636723 | *C3* |
| rs344548 | intron | 19 | 6636817 | *C3* |
| rs7257062 | intron | 19 | 6636945 | *C3* |
| rs2241392 | intron | 19 | 6636983 | *C3* |
| rs237554 | intron | 19 | 6637659 | *C3* |
| rs344546 | intron | 19 | 6638132 | *C3* |
| rs344545 | intron | 19 | 6638142 | *C3* |
| rs344544 | intron | 19 | 6638148 | *C3* |
| rs344543 | intron | 19 | 6638414 | *C3* |
| rs344542 | intron | 19 | 6638517 | *C3* |
| rs11569523 | intron | 19 | 6640042 | *C3* |
| rs11569514 | intron | 19 | 6641276 | *C3* |
| rs3745568 | intron | 19 | 6641613 | *C3* |
| rs428453 | synonymous | 19 | 6653157 | *C3* |
| rs432823 | intron | 19 | 6653246 | *C3* |
| rs11569450 | intron | 19 | 6653455 | *C3* |
| rs406514 | intron | 19 | 6653598 | *C3* |
| rs11672613 | intron | 19 | 6656246 | *C3* |
| rs8112351 | intron | 19 | 6657298 | *C3* |
| rs11569429 | intron | 19 | 6660074 | *C3* |
| rs2230205 | synonymous | 19 | 6660704 | *C3* |
| rs2230204 | synonymous | 19 | 6660848 | *C3* |
| rs2230203 | synonymous | 19 | 6661782 | *C3* |
| rs1047286 | nonsynonymous | 19 | 6664262 | *C3* |
| rs2230199 | nonsynonymous | 19 | 6669387 | *C3* |
| rs2250656 | intron | 19 | 6669534 | *C3* |
| rs11671717 | NA | 19 | 52500638 | intergenic |
| rs7492 | UTR-3 | 20 | 23008257 | *CD93* |
| rs2749812 | UTR-3 | 20 | 23010927 | *CD93* |
| rs2749813 | UTR-3 | 20 | 23010984 | *CD93* |
| rs3746731 | nonsynonymous | 20 | 23013209 | *CD93* |
| rs3746732 | synonymous | 20 | 23013342 | *CD93* |
| rs1048118 | synonymous | X | 47368744 | *CFP* |
| rs8177068 | nonsynonymous | X | 47373937 | *CFP* |
| rs5964488 | intron | X | 65170494 | *VSIG4* |

**Supplementary table 2**. Association results of SNPs genotyped over the complement genes within Newcastle cohort.

| **SNP** | **Ch** | **Position** | **Gene** | **Alleles** | **MAF** | **Chi-squared statistics** | **P-value** |
| --- | --- | --- | --- | --- | --- | --- | --- |
| rs2761434 | 1 | 205984122 | intergenic CR1L-CD46 | A/G | 0.26 | 17.39 | 3.05E-05 |
| rs2796275 | 1 | 206017113 | *CD46* | C/T | 0.35 | 15.05 | 1.05E-04 |
| rs10449303 | 1 | 206027681 | *CD46* | G/A | 0.41 | 11.87 | 5.70E-04 |
| rs7144 | 1 | 206034342 | *CD46* | C/T | 0.41 | 11.72 | 6.17E-04 |
| rs2796278 | 1 | 206022446 | *CD46* | C/A | 0.46 | 10.97 | 9.25E-04 |
| rs3753396 | 1 | 194962365 | *CFH* | G/A | 0.19 | 10.28 | 1.35E-03 |
| rs1065489 | 1 | 194976397 | *CFH* | T/G | 0.19 | 9.96 | 1.60E-03 |
| rs3795341 | 1 | 195153897 | *CFHR4* | T/C | 0.20 | 9.44 | 2.13E-03 |
| rs424535 | 1 | 194975846 | *CFH* | T/A | 0.41 | 8.71 | 3.16E-03 |
| rs1329423 | 1 | 194913010 | *CFH* | G/A | 0.27 | 8.62 | 3.33E-03 |
| rs9427934 | 1 | 195189483 | *CFHR2* | A/G | 0.35 | 8.18 | 4.24E-03 |
| rs12405238 | 1 | 194928236 | *CFH* | T/G | 0.27 | 8.16 | 4.28E-03 |
| rs9937837 | 16 | 31206440 | *CD11b* | G/T | 0.28 | 7.57 | 5.95E-03 |
| rs7499077 | 16 | 31225006 | *CD11b* | A/G | 0.28 | 7.57 | 5.95E-03 |
| rs2250656 | 19 | 6669534 | *C3* | G/A | 0.27 | 6.45 | 1.11E-02 |
| rs800292 | 1 | 194908856 | *CFH* | T/C | 0.21 | 5.65 | 1.74E-02 |
| rs17044576 | 1 | 205701293 | *CR2* | C/T | 0.04 | 5.57 | 1.83E-02 |
| rs11569429 | 19 | 6660074 | *C3* | A/G | 0.15 | 5.08 | 2.43E-02 |
| rs6680396 | 1 | 194899093 | *CFH* | G/A | 0.20 | 4.82 | 2.81E-02 |
| rs11861251 | 16 | 31196897 | *CD11b* | C/T | 0.17 | 4.74 | 2.95E-02 |
| rs2796269 | 1 | 206003509 | *CD46* | T/C | 0.34 | 4.64 | 3.12E-02 |
| rs10127904 | 1 | 205814730 | *CR1* | G/T | 0.35 | 4.32 | 3.77E-02 |
| rs7416336 | 1 | 195138798 | *CFHR4* | G/C | 0.20 | 4.30 | 3.81E-02 |
| rs7555070 | 1 | 195248777 | intergenic CFHR5-F13B | C/T | 0.39 | 4.25 | 3.92E-02 |
| rs4698788 | 4 | 110938983 | *CFI* | C/T | 0.03 | 4.23 | 3.98E-02 |
| rs850313 | 3 | 188435920 | *MASP1* | G/C | 0.28 | 4.12 | 4.23E-02 |
| rs6661764 | 1 | 205875008 | *CR1* | G/C | 0.26 | 4.11 | 4.26E-02 |
| rs1831150 | 1 | 205737061 | *CR1* | G/A | 0.34 | 4.03 | 4.46E-02 |
| rs1576340 | 1 | 194965334 | *CFH* | A/C | 0.19 | 3.95 | 4.69E-02 |
| rs7951 | 19 | 6632991 | *C3* | T/C | 0.08 | 3.86 | 4.94E-02 |
| rs2025935 | 1 | 205742278 | *CR1* | T/C | 0.35 | 3.79 | 5.16E-02 |
| rs3733003 | 3 | 188421885 | *MASP1* | A/T | 0.24 | 3.75 | 5.30E-02 |
| rs11032349 | 11 | 33686248 | *CD59* | T/A | 0.10 | 3.69 | 5.46E-02 |
| rs9332730 | 6 | 32019988 | *CFB/C2* | C/G | 0.05 | 3.68 | 5.51E-02 |
| rs7545083 | 1 | 205362511 | *C4BPA* | C/T | 0.33 | 3.55 | 5.97E-02 |
| rs2002665 | 3 | 188434166 | *MASP1* | A/G | 0.29 | 3.47 | 6.26E-02 |
| rs850316 | 3 | 188430846 | *MASP1* | T/C | 0.46 | 3.46 | 6.29E-02 |
| rs7842 | 12 | 8102454 | *C3AR1* | G/A | 0.31 | 3.44 | 6.35E-02 |
| rs4425986 | 1 | 205360403 | *C4BPA* | C/T | 0.43 | 3.39 | 6.54E-02 |
| rs4915559 | 1 | 195153393 | *CFHR4* | C/T | 0.21 | 3.33 | 6.80E-02 |
| rs4844573 | 1 | 205371523 | *C4BPA* | C/T | 0.35 | 3.26 | 7.08E-02 |
| rs3914010 | 3 | 188422267 | *MASP1* | G/A | 0.13 | 3.20 | 7.37E-02 |
| rs3748557 | 1 | 195213492 | *CFHR5* | T/A | 0.26 | 3.05 | 8.06E-02 |
| rs12740591 | 1 | 22837590 | *C1QA* | T/C | 0.05 | 3.00 | 8.31E-02 |
| rs324056 | 5 | 40968997 | *C7* | T/G | 0.15 | 2.99 | 8.38E-02 |
| rs3737002 | 1 | 205827396 | *CR1* | T/C | 0.25 | 2.93 | 8.67E-02 |
| rs7851696 | 9 | 136918912 | *FCN2* | T/G | 0.12 | 2.92 | 8.74E-02 |
| rs11150610 | 16 | 31241737 | *CD11b* | A/C | 0.39 | 2.87 | 9.00E-02 |
| rs17220750 | 9 | 122827820 | *C5* | A/G | 0.11 | 2.77 | 9.61E-02 |
| rs7524776 | 1 | 194889960 | *CFH* | C/T | 0.16 | 2.76 | 9.65E-02 |
| rs3792642 | 5 | 40968910 | *C7* | T/C | 0.18 | 2.71 | 9.96E-02 |
| rs7954916 | 12 | 8109659 | *C3AR1* | A/C | 0.29 | 2.65 | 1.03E-01 |
| rs3815623 | 3 | 188441736 | *MASP1* | T/C | 0.46 | 2.54 | 1.11E-01 |
| rs2296160 | 1 | 205861943 | *CR1* | T/C | 0.20 | 2.51 | 1.13E-01 |
| rs12032512 | 1 | 205719801 | *CR2* | G/C | 0.49 | 2.49 | 1.15E-01 |
| rs2230205 | 19 | 6660704 | *C3* | A/G | 0.14 | 2.46 | 1.17E-01 |
| rs791587 | 10 | 6128705 | *IL2RA* | G/A | 0.42 | 2.32 | 1.28E-01 |
| rs17021100 | 1 | 205360630 | *C4BPA* | C/G | 0.12 | 2.31 | 1.28E-01 |
| rs10922102 | 1 | 194934910 | *CFH* | C/T | 0.36 | 2.31 | 1.28E-01 |
| rs395544 | 1 | 194964895 | *CFH* | T/C | 0.40 | 2.27 | 1.32E-01 |
| rs10905668 | 10 | 6132061 | *IL2RA* | T/C | 0.24 | 2.27 | 1.32E-01 |
| rs17259045 | 1 | 205849330 | *CR1* | G/A | 0.13 | 2.25 | 1.34E-01 |
| rs2491393 | 1 | 205366882 | *C4BPA* | G/A | 0.36 | 2.25 | 1.34E-01 |
| rs263275 | 5 | 39350934 | *C9* | T/G | 0.49 | 2.22 | 1.36E-01 |
| rs6683663 | 1 | 57129064 | *C8A* | G/A | 0.14 | 2.21 | 1.37E-01 |
| rs698090 | 3 | 188446994 | *MASP1* | G/A | 0.36 | 2.20 | 1.38E-01 |
| rs1061170 | 1 | 194925860 | *CFH* | C/T | 0.36 | 2.16 | 1.42E-01 |
| rs7529589 | 1 | 194924902 | *CFH* | T/C | 0.36 | 2.13 | 1.45E-01 |
| rs11645653 | 16 | 31220356 | *CD11b* | C/T | 0.23 | 2.10 | 1.47E-01 |
| rs12116643 | 1 | 195239806 | *CFHR5* | C/T | 0.13 | 2.09 | 1.49E-01 |
| rs12685659 | 9 | 136916540 | *FCN2* | T/A | 0.11 | 2.04 | 1.53E-01 |
| rs2300932 | 9 | 22810257 | *C5* | A/C | 0.41 | 1.95 | 1.62E-01 |
| rs2236217 | 1 | 57203393 | *C8B* | T/G | 0.11 | 1.93 | 1.65E-01 |
| rs344546 | 19 | 6638132 | *C3* | T/C | 0.37 | 1.91 | 1.67E-01 |
| rs867231 | 8 | 27523309 | *CLU* | G/C | 0.27 | 1.91 | 1.67E-01 |
| rs6699859 | 1 | 57133038 | *C8A* | T/C | 0.31 | 1.90 | 1.68E-01 |
| rs344545 | 19 | 6638142 | *C3* | T/C | 0.37 | 1.90 | 1.69E-01 |
| rs324058 | 5 | 40997651 | *C7* | G/T | 0.27 | 1.86 | 1.73E-01 |
| rs6602391 | 10 | 6118038 | *IL2RA* | A/G | 0.05 | 1.85 | 1.73E-01 |
| rs3774279 | 3 | 188463795 | *MASP1* | G/A | 0.36 | 1.85 | 1.73E-01 |
| rs6875226 | 5 | 40979199 | *C7* | C/T | 0.18 | 1.85 | 1.74E-01 |
| rs344543 | 19 | 6638414 | *C3* | C/G | 0.37 | 1.82 | 1.77E-01 |
| rs344542 | 19 | 6638517 | *C3* | C/T | 0.37 | 1.82 | 1.77E-01 |
| rs9200 | 5 | 41178363 | *C6* | A/G | 0.47 | 1.82 | 1.78E-01 |
| rs12658133 | 5 | 40968376 | *C7* | G/A | 0.20 | 1.80 | 1.79E-01 |
| rs4571969 | 1 | 205372211 | *C4BPA* | T/C | 0.22 | 1.80 | 1.80E-01 |
| rs324062 | 5 | 40956290 | *C7* | A/C | 0.19 | 1.77 | 1.84E-01 |
| rs514943 | 1 | 194930536 | *CFH* | G/A | 0.36 | 1.76 | 1.85E-01 |
| rs2455313 | 5 | 40982362 | *C7* | A/T | 0.18 | 1.75 | 1.86E-01 |
| rs1143683 | 16 | 31244389 | *CD11b* | T/C | 0.13 | 1.72 | 1.89E-01 |
| rs3864099 | 3 | 188481632 | *MASP1* | G/T | 0.32 | 1.70 | 1.92E-01 |
| rs7533852 | 1 | 205562050 | *CD55* | T/G | 0.45 | 1.70 | 1.92E-01 |
| rs1143678 | 16 | 31250506 | *CD11b* | T/C | 0.13 | 1.69 | 1.93E-01 |
| rs9888879 | 16 | 31217873 | *CD11b* | C/T | 0.11 | 1.69 | 1.94E-01 |
| rs3843010 | 3 | 188419741 | *MASP1* | T/G | 0.38 | 1.66 | 1.97E-01 |
| rs2039544 | 1 | 205486540 | intergenic | T/G | 0.45 | 1.62 | 2.03E-01 |
| rs13089330 | 3 | 188492964 | *MASP1* | T/C | 0.33 | 1.62 | 2.04E-01 |
| rs7193268 | 16 | 31248498 | *CD11b* | T/C | 0.13 | 1.60 | 2.06E-01 |
| rs1800450 | 10 | 54201241 | *MBL2* | A/G | 0.14 | 1.59 | 2.07E-01 |
| rs7732104 | 5 | 41019193 | *C7* | A/G | 0.17 | 1.59 | 2.07E-01 |
| rs11959122 | 5 | 39346359 | *C9* | T/C | 0.12 | 1.58 | 2.09E-01 |
| rs11256497 | 10 | 6127800 | *IL2RA* | A/G | 0.32 | 1.54 | 2.15E-01 |
| rs11206932 | 1 | 57134595 | *C8A* | A/T | 0.12 | 1.51 | 2.19E-01 |
| rs710462 | 3 | 188445824 | *MASP1* | T/G | 0.46 | 1.51 | 2.19E-01 |
| rs4837805 | 9 | 122786076 | *C5* | G/A | 0.40 | 1.50 | 2.20E-01 |
| rs6565228 | 16 | 31236781 | *CD11b* | A/G | 0.11 | 1.46 | 2.27E-01 |
| rs10922096 | 1 | 194929082 | *CFH* | T/C | 0.46 | 1.44 | 2.30E-01 |
| rs12722596 | 10 | 6096300 | *IL2RA* | G/A | 0.10 | 1.43 | 2.32E-01 |
| rs850317 | 3 | 188430495 | *MASP1* | A/G | 0.36 | 1.42 | 2.34E-01 |
| rs2455308 | 5 | 40965270 | *C7* | T/C | 0.20 | 1.41 | 2.35E-01 |
| rs7135975 | 12 | 7134709 | *CIR* | G/A | 0.27 | 1.40 | 2.37E-01 |
| rs17040 | 3 | 188420262 | *MASP1* | T/C | 0.44 | 1.39 | 2.38E-01 |
| rs1143664 | 12 | 7045308 | *C1S* | G/A | 0.06 | 1.35 | 2.46E-01 |
| rs918608 | 5 | 41188446 | *C6* | C/T | 0.20 | 1.34 | 2.48E-01 |
| rs3118470 | 10 | 6141719 | *IL2RA* | C/T | 0.30 | 1.33 | 2.48E-01 |
| rs11596355 | 10 | 6104187 | *IL2RA* | C/T | 0.10 | 1.33 | 2.49E-01 |
| rs12377780 | 9 | 136948782 | *FCN1* | A/C | 0.31 | 1.26 | 2.61E-01 |
| rs10922106 | 1 | 194958087 | *CFH* | G/A | 0.42 | 1.25 | 2.64E-01 |
| rs706780 | 10 | 6127032 | *IL2RA* | A/C | 0.04 | 1.23 | 2.68E-01 |
| rs835209 | 5 | 39386120 | *C9* | T/C | 0.03 | 1.21 | 2.72E-01 |
| rs419137 | 1 | 194963498 | *CFH* | C/A | 0.10 | 1.20 | 2.73E-01 |
| rs6697228 | 1 | 57122052 | *C8A* | C/T | 0.13 | 1.19 | 2.75E-01 |
| rs17186575 | 1 | 205351774 | *C4BPA* | A/G | 0.07 | 1.18 | 2.77E-01 |
| rs1652333 | 1 | 205537083 | intergenic *C4BPA-CR1* | C/T | 0.28 | 1.18 | 2.78E-01 |
| rs14374 | 1 | 206034880 | *CD46* | C/T | 0.05 | 1.17 | 2.80E-01 |
| rs6695321 | 1 | 194942484 | *CFH* | G/A | 0.39 | 1.16 | 2.81E-01 |
| rs4915309 | 1 | 170174251 | intergenic | A/T | 0.12 | 1.16 | 2.81E-01 |
| rs1048709 | 6 | 32022914 | *CFB* | A/G | 0.18 | 1.15 | 2.83E-01 |
| rs2104286 | 10 | 6139051 | *IL2RA* | G/A | 0.32 | 1.15 | 2.84E-01 |
| rs6694643 | 1 | 57121995 | *C8A* | T/A | 0.13 | 1.14 | 2.85E-01 |
| rs619545 | 1 | 57145179 | *C8A* | G/A | 0.27 | 1.14 | 2.85E-01 |
| rs6686359 | 1 | 57129168 | *C8A* | C/T | 0.02 | 1.14 | 2.87E-01 |
| rs3792640 | 5 | 40968595 | *C7* | A/G | 0.20 | 1.13 | 2.89E-01 |
| rs17057444 | 8 | 27509710 | *CLU* | G/C | 0.03 | 1.12 | 2.91E-01 |
| rs9880761 | 3 | 188423991 | *MASP1* | G/A | 0.33 | 1.11 | 2.92E-01 |
| rs7308467 | 12 | 7134924 | *CIR* | C/T | 0.32 | 1.08 | 2.99E-01 |
| rs6685931 | 1 | 195133856 | *CFHR4* | C/T | 0.38 | 1.08 | 3.00E-01 |
| rs9429944 | 1 | 205736930 | *CR1* | T/A | 0.23 | 1.07 | 3.00E-01 |
| rs12087872 | 1 | 205373156 | *C4BPA* | G/A | 0.23 | 1.07 | 3.02E-01 |
| rs12722486 | 10 | 6143768 | *IL2RA* | A/G | 0.04 | 1.05 | 3.05E-01 |
| rs12116668 | 1 | 57143721 | *C8A* | C/T | 0.11 | 1.05 | 3.05E-01 |
| rs17258982 | 1 | 205719987 | *CR2* | G/A | 0.08 | 1.04 | 3.07E-01 |
| rs10922153 | 1 | 195245238 | *CFHR5* | G/T | 0.48 | 1.02 | 3.13E-01 |
| rs10818495 | 9 | 122797008 | *C5* | A/C | 0.47 | 1.02 | 3.13E-01 |
| rs1774900 | 1 | 57149737 | *C8A* | C/G | 0.05 | 1.02 | 3.13E-01 |
| rs3774275 | 3 | 188447962 | *MASP1* | G/A | 0.29 | 1.01 | 3.15E-01 |
| rs662377 | 1 | 57097100 | *C8A* | T/C | 0.43 | 0.97 | 3.26E-01 |
| rs2230203 | 19 | 6661782 | *C3* | A/C | 0.16 | 0.96 | 3.28E-01 |
| rs291990 | 1 | 22852242 | *C1QB* | A/C | 0.45 | 0.94 | 3.32E-01 |
| rs2274037 | 10 | 6102114 | *IL2RA* | A/G | 0.03 | 0.94 | 3.33E-01 |
| rs1389623 | 19 | 6635197 | *C3* | T/C | 0.12 | 0.94 | 3.33E-01 |
| rs13064994 | 3 | 188488710 | *MASP1* | A/G | 0.27 | 0.93 | 3.35E-01 |
| rs2989725 | 9 | 136947826 | *FCN1* | C/A | 0.32 | 0.92 | 3.37E-01 |
| rs1853883 | 1 | 195148223 | *CFHR4* | C/G | 0.43 | 0.90 | 3.44E-01 |
| rs3805713 | 5 | 41189118 | *C6* | A/T | 0.37 | 0.89 | 3.46E-01 |
| rs17301146 | 1 | 57172849 | *C8B* | A/G | 0.16 | 0.89 | 3.46E-01 |
| rs11064498 | 12 | 7041768 | *C1S* | G/A | 0.16 | 0.88 | 3.47E-01 |
| rs9429945 | 1 | 205743391 | *CR1* | T/C | 0.20 | 0.88 | 3.49E-01 |
| rs11569514 | 19 | 6641276 | *C3* | T/A | 0.13 | 0.88 | 3.49E-01 |
| rs3746732 | 20 | 23013342 | *CD93* | A/G | 0.24 | 0.88 | 3.49E-01 |
| rs3128626 | 9 | 136913377 | *FCN2* | T/C | 0.28 | 0.87 | 3.51E-01 |
| rs4317805 | 1 | 205719615 | *CR2* | A/G | 0.08 | 0.86 | 3.53E-01 |
| rs706778 | 10 | 6138955 | *IL2RA* | A/G | 0.38 | 0.86 | 3.53E-01 |
| rs6691117 | 1 | 205849554 | *CR1* | G/A | 0.18 | 0.85 | 3.55E-01 |
| rs823940 | 3 | 188462698 | *MASP1* | G/A | 0.08 | 0.85 | 3.57E-01 |
| rs683916 | 1 | 57198062 | *C8B* | A/T | 0.33 | 0.85 | 3.58E-01 |
| rs1126618 | 1 | 205364303 | *C4BPA* | T/C | 0.17 | 0.84 | 3.59E-01 |
| rs10512764 | 5 | 41193097 | *C6* | T/C | 0.15 | 0.82 | 3.65E-01 |
| rs7024491 | 9 | 136914905 | *FCN2* | G/A | 0.38 | 0.82 | 3.66E-01 |
| rs17030 | 19 | 6628989 | *C3* | G/A | 0.47 | 0.80 | 3.70E-01 |
| rs12021671 | 1 | 205724768 | *CR2* | A/G | 0.34 | 0.80 | 3.71E-01 |
| rs2277984 | 19 | 6630511 | *C3* | G/A | 0.48 | 0.80 | 3.72E-01 |
| rs344555 | 19 | 6630360 | *C3* | A/G | 0.20 | 0.78 | 3.77E-01 |
| rs11953839 | 5 | 39365195 | *C9* | A/G | 0.40 | 0.78 | 3.78E-01 |
| rs4521835 | 9 | 136919127 | *FCN2* | G/T | 0.42 | 0.77 | 3.81E-01 |
| rs6822976 | 4 | 110885741 | *CFI* | A/G | 0.49 | 0.77 | 3.82E-01 |
| rs696760 | 5 | 39369251 | *C9* | C/G | 0.11 | 0.75 | 3.86E-01 |
| rs2191959 | 9 | 122723655 | *TRAF1* | A/T | 0.08 | 0.74 | 3.89E-01 |
| rs2405237 | 1 | 57113134 | *C8A* | A/G | 0.04 | 0.74 | 3.90E-01 |
| rs4597342 | 16 | 31251270 | *CD11b* | T/C | 0.32 | 0.74 | 3.90E-01 |
| rs7026551 | 9 | 122772954 | *C5* | C/A | 0.20 | 0.73 | 3.93E-01 |
| rs6602392 | 10 | 6118085 | *IL2RA* | A/C | 0.09 | 0.72 | 3.95E-01 |
| rs344548 | 19 | 6636817 | *C3* | C/G | 0.17 | 0.72 | 3.95E-01 |
| rs7746553 | 6 | 32003952 | *C2* | G/C | 0.14 | 0.70 | 4.01E-01 |
| rs17611 | 9 | 122809021 | *C5* | A/G | 0.42 | 0.70 | 4.02E-01 |
| rs155377 | 5 | 39344808 | *C9* | C/T | 0.44 | 0.70 | 4.03E-01 |
| rs17201103 | 5 | 41221403 | *C6* | T/C | 0.31 | 0.69 | 4.06E-01 |
| rs344544 | 19 | 6638148 | *C3* | T/G | 0.13 | 0.68 | 4.10E-01 |
| rs2749813 | 20 | 23010984 | *CD93* | T/C | 0.33 | 0.67 | 4.13E-01 |
| rs1571344 | 1 | 205737551 | *CR1* | C/T | 0.10 | 0.66 | 4.16E-01 |
| rs3749519 | 5 | 41017132 | *C7* | G/A | 0.37 | 0.66 | 4.18E-01 |
| rs294185 | 1 | 22844479 | *C1QC* | C/T | 0.37 | 0.65 | 4.20E-01 |
| rs700233 | 5 | 39400311 | *C9* | A/G | 0.40 | 0.64 | 4.25E-01 |
| rs11256456 | 10 | 6120718 | *IL2RA* | C/T | 0.22 | 0.63 | 4.26E-01 |
| rs17020983 | 1 | 205354920 | *C4BPA* | T/C | 0.06 | 0.63 | 4.29E-01 |
| rs11569538 | 19 | 6636723 | *C3* | C/G | 0.08 | 0.62 | 4.30E-01 |
| rs2274567 | 1 | 205820244 | *CR1* | G/A | 0.15 | 0.62 | 4.30E-01 |
| rs1047286 | 19 | 6664262 | *C3* | T/C | 0.19 | 0.60 | 4.39E-01 |
| rs4469075 | 4 | 110910035 | *CFI* | C/G | 0.26 | 0.60 | 4.40E-01 |
| rs6860770 | 5 | 41221669 | *C6* | C/A | 0.10 | 0.59 | 4.44E-01 |
| rs3128627 | 9 | 136912613 | *FCN2* | G/A | 0.08 | 0.57 | 4.49E-01 |
| rs25681 | 9 | 122819826 | *C5* | T/C | 0.42 | 0.57 | 4.50E-01 |
| rs16910280 | 9 | 122844597 | *C5* | T/C | 0.15 | 0.56 | 4.54E-01 |
| rs2241394 | 19 | 6636230 | *C3* | G/C | 0.12 | 0.55 | 4.59E-01 |
| rs237554 | 19 | 6637659 | *C3* | A/G | 0.14 | 0.54 | 4.61E-01 |
| rs292001 | 1 | 22837545 | *C1QA* | A/G | 0.37 | 0.54 | 4.61E-01 |
| rs4077810 | 16 | 31248410 | *CD11b* | T/C | 0.32 | 0.54 | 4.63E-01 |
| rs2269066 | 9 | 122776839 | *C5* | T/C | 0.09 | 0.54 | 4.63E-01 |
| rs10473238 | 5 | 41231987 | *C6* | T/C | 0.10 | 0.52 | 4.71E-01 |
| rs17375194 | 3 | 188470176 | *MASP1* | G/C | 0.13 | 0.51 | 4.75E-01 |
| rs9331930 | 8 | 27514211 | *CLU* | C/A | 0.28 | 0.51 | 4.76E-01 |
| rs7040033 | 9 | 122798865 | *C5* | A/G | 0.43 | 0.51 | 4.77E-01 |
| rs942201 | 10 | 6126298 | *IL2RA* | T/G | 0.24 | 0.51 | 4.77E-01 |
| rs7183 | 12 | 7048280 | *C1S* | T/G | 0.14 | 0.50 | 4.80E-01 |
| rs9998151 | 4 | 110886794 | *CFI* | C/T | 0.03 | 0.49 | 4.85E-01 |
| rs7041446 | 9 | 136915875 | *FCN2* | G/A | 0.45 | 0.49 | 4.86E-01 |
| rs2511989 | 11 | 57134901 | *SERPING1* | A/G | 0.40 | 0.48 | 4.88E-01 |
| rs1001073 | 3 | 188427399 | *MASP1* | G/A | 0.30 | 0.48 | 4.89E-01 |
| rs614709 | 1 | 205819898 | *CR1* | C/T | 0.18 | 0.48 | 4.90E-01 |
| rs698092 | 3 | 188452328 | *MASP1* | G/A | 0.35 | 0.47 | 4.91E-01 |
| rs2675982 | 5 | 40996035 | *C7* | A/G | 0.05 | 0.46 | 4.96E-01 |
| rs4151667 | 6 | 32022003 | *CFB* | A/T | 0.04 | 0.46 | 4.99E-01 |
| rs6848178 | 4 | 110885620 | *CFI* | T/A | 0.42 | 0.45 | 5.00E-01 |
| rs1048971 | 1 | 205712945 | *CR2* | A/G | 0.38 | 0.45 | 5.01E-01 |
| rs3745568 | 19 | 6641613 | *C3* | C/A | 0.13 | 0.44 | 5.06E-01 |
| rs3774268 | 3 | 188437018 | *MASP1* | A/G | 0.13 | 0.44 | 5.07E-01 |
| rs874603 | 3 | 188435731 | *MASP1* | G/A | 0.08 | 0.44 | 5.08E-01 |
| rs1357134 | 3 | 188462462 | *MASP1* | G/A | 0.39 | 0.44 | 5.08E-01 |
| rs17040805 | 4 | 110884246 | *CFI* | A/C | 0.06 | 0.44 | 5.09E-01 |
| rs537160 | 6 | 32024379 | *CFB* | T/C | 0.34 | 0.44 | 5.09E-01 |
| rs17468519 | 5 | 39339216 | *C9* | A/G | 0.08 | 0.43 | 5.10E-01 |
| rs17687664 | 11 | 33696988 | *CD59* | C/G | 0.17 | 0.43 | 5.13E-01 |
| rs11136000 | 8 | 27520436 | *CLU* | T/C | 0.40 | 0.42 | 5.17E-01 |
| rs3181274 | 11 | 33711865 | *CD59* | A/G | 0.36 | 0.41 | 5.22E-01 |
| rs791590 | 10 | 6130328 | *IL2RA* | T/A | 0.19 | 0.41 | 5.23E-01 |
| rs2072633 | 6 | 32027557 | *CFB* | T/C | 0.44 | 0.40 | 5.26E-01 |
| rs2025345 | 10 | 6107694 | *IL2RA* | G/A | 0.36 | 0.40 | 5.27E-01 |
| rs1887632 | 1 | 205738244 | *CR1* | A/G | 0.07 | 0.40 | 5.28E-01 |
| rs11721403 | 4 | 110913018 | *CFI* | G/A | 0.38 | 0.39 | 5.30E-01 |
| rs638919 | 1 | 57149760 | *C8A* | G/A | 0.33 | 0.39 | 5.33E-01 |
| rs1035497 | 5 | 41205972 | *C6* | G/A | 0.15 | 0.39 | 5.35E-01 |
| rs2228150 | 10 | 6107975 | *IL2RA* | A/G | 0.02 | 0.38 | 5.36E-01 |
| rs12614 | 6 | 32022158 | *CFB* | T/C | 0.10 | 0.38 | 5.36E-01 |
| rs6602398 | 10 | 6122959 | *IL2RA* | T/G | 0.30 | 0.38 | 5.36E-01 |
| rs16840522 | 1 | 194977539 | *CFH* | C/T | 0.22 | 0.38 | 5.39E-01 |
| rs2300955 | 1 | 57116750 | *C8A* | T/C | 0.18 | 0.38 | 5.39E-01 |
| rs17186582 | 1 | 205352254 | *C4BPA* | A/C | 0.31 | 0.38 | 5.40E-01 |
| rs11256433 | 10 | 6117851 | *IL2RA* | G/T | 0.22 | 0.38 | 5.40E-01 |
| rs6665881 | 1 | 205723228 | *CR2* | C/G | 0.41 | 0.36 | 5.50E-01 |
| rs7519492 | 1 | 195106748 | intergenic CFHR1-CFHR4 | C/T | 0.17 | 0.35 | 5.53E-01 |
| rs9332739 | 6 | 32011783 | *C2* | C/G | 0.05 | 0.35 | 5.53E-01 |
| rs7519408 | 1 | 205727912 | *CR2* | G/C | 0.13 | 0.35 | 5.55E-01 |
| rs1474891 | 1 | 11004467 | *TARDBP* | C/A | 0.18 | 0.35 | 5.56E-01 |
| rs16861893 | 3 | 188491870 | *MASP1* | A/G | 0.07 | 0.34 | 5.59E-01 |
| rs2273121 | 11 | 33714346 | *CD59* | T/C | 0.26 | 0.34 | 5.60E-01 |
| rs658285 | 1 | 57188923 | *C8B* | T/C | 0.31 | 0.34 | 5.61E-01 |
| rs1423394 | 5 | 41152600 | intergenic | C/T | 0.30 | 0.33 | 5.64E-01 |
| rs599857 | 1 | 57201177 | *C8B* | C/T | 0.26 | 0.33 | 5.64E-01 |
| rs11569450 | 19 | 6653455 | *C3* | G/C | 0.11 | 0.33 | 5.67E-01 |
| rs2071006 | 9 | 138959725 | *C8G* | T/G | 0.50 | 0.33 | 5.68E-01 |
| rs11569523 | 19 | 6640042 | *C3* | A/G | 0.06 | 0.32 | 5.69E-01 |
| rs2842704 | 1 | 205356059 | *C4BPA* | G/A | 0.14 | 0.31 | 5.78E-01 |
| rs2241392 | 19 | 6636983 | *C3* | G/C | 0.34 | 0.30 | 5.81E-01 |
| rs292010 | 1 | 22862332 | intergenic | A/G | 0.06 | 0.29 | 5.90E-01 |
| rs7257062 | 19 | 6636945 | *C3* | T/C | 0.42 | 0.29 | 5.91E-01 |
| rs9663421 | 10 | 6095610 | *IL2RA* | T/C | 0.27 | 0.29 | 5.92E-01 |
| rs2842707 | 1 | 205364107 | *C4BPA* | C/G | 0.42 | 0.28 | 5.96E-01 |
| rs10824794 | 10 | 54203195 | *MBL2* | T/A | 0.23 | 0.28 | 5.97E-01 |
| rs710473 | 3 | 188475684 | *MASP1* | G/A | 0.15 | 0.28 | 5.98E-01 |
| rs3864098 | 3 | 188478554 | *MASP1* | C/T | 0.15 | 0.28 | 5.98E-01 |
| rs12034383 | 1 | 205870218 | *CR1* | G/A | 0.37 | 0.27 | 6.00E-01 |
| rs6690215 | 1 | 205722673 | *CR2* | C/T | 0.42 | 0.27 | 6.00E-01 |
| rs12734030 | 1 | 205860587 | *CR1* | T/C | 0.14 | 0.27 | 6.01E-01 |
| rs831628 | 11 | 33701421 | *CD59* | G/C | 0.21 | 0.27 | 6.02E-01 |
| rs647571 | 1 | 57192195 | *C8B* | A/G | 0.33 | 0.27 | 6.05E-01 |
| rs12711521 | 1 | 11013503 | *MASP2* | C/A | 0.18 | 0.26 | 6.08E-01 |
| rs16910255 | 9 | 122796804 | *C5* | T/C | 0.10 | 0.26 | 6.13E-01 |
| rs867230 | 8 | 27524420 | *CLU* | C/A | 0.41 | 0.25 | 6.20E-01 |
| rs831636 | 11 | 33696174 | *CD59* | T/C | 0.32 | 0.25 | 6.20E-01 |
| rs12153063 | 5 | 40943951 | *C7* | A/C | 0.10 | 0.25 | 6.20E-01 |
| rs1877134 | 10 | 54184769 | intergenic | G/A | 0.40 | 0.24 | 6.24E-01 |
| rs1423396 | 5 | 41158467 | intergenic | G/A | 0.19 | 0.24 | 6.26E-01 |
| rs850312 | 3 | 188436502 | *MASP1* | A/G | 0.34 | 0.23 | 6.30E-01 |
| rs1468673 | 9 | 122849711 | *C5* | C/T | 0.43 | 0.23 | 6.30E-01 |
| rs13157656 | 5 | 41000609 | *C7* | C/A | 0.25 | 0.23 | 6.31E-01 |
| rs12107082 | 3 | 188439068 | *MASP1* | A/G | 0.04 | 0.23 | 6.34E-01 |
| rs1105323 | 9 | 136942234 | *FCN1* | A/G | 0.39 | 0.23 | 6.34E-01 |
| rs11951782 | 5 | 41227310 | *C6* | A/T | 0.12 | 0.22 | 6.36E-01 |
| rs12073436 | 1 | 22843495 | *C1QC* | T/C | 0.08 | 0.22 | 6.37E-01 |
| rs7027797 | 9 | 122811620 | *C5* | C/T | 0.13 | 0.22 | 6.39E-01 |
| rs17760306 | 11 | 33694612 | *CD59* | G/T | 0.16 | 0.22 | 6.39E-01 |
| rs594661 | 1 | 57174570 | *C8B* | A/G | 0.08 | 0.22 | 6.42E-01 |
| rs3107217 | 3 | 188479513 | *MASP1* | T/A | 0.11 | 0.22 | 6.43E-01 |
| rs4403634 | 1 | 205334714 | *C4BPB* | C/A | 0.46 | 0.21 | 6.43E-01 |
| rs2386841 | 10 | 6097738 | *IL2RA* | A/C | 0.13 | 0.21 | 6.45E-01 |
| rs698093 | 3 | 188452845 | *MASP1* | T/G | 0.22 | 0.21 | 6.46E-01 |
| rs704697 | 11 | 33681356 | *CD59* | T/C | 0.44 | 0.21 | 6.46E-01 |
| rs10818494 | 9 | 122786259 | *C5* | C/T | 0.40 | 0.21 | 6.50E-01 |
| rs12136082 | 1 | 11024862 | *MASP2* | A/G | 0.20 | 0.20 | 6.52E-01 |
| rs11064497 | 12 | 7039922 | *C1S* | T/C | 0.15 | 0.20 | 6.52E-01 |
| rs8048583 | 16 | 31187037 | *CD11b* | T/C | 0.35 | 0.20 | 6.54E-01 |
| rs11957341 | 5 | 41233229 | *C6* | A/C | 0.43 | 0.20 | 6.56E-01 |
| rs7671905 | 4 | 110942335 | *CFI* | T/C | 0.29 | 0.20 | 6.56E-01 |
| rs2989735 | 9 | 136941237 | *FCN1* | T/C | 0.39 | 0.20 | 6.56E-01 |
| rs7492 | 20 | 23008257 | *CD93* | A/G | 0.09 | 0.20 | 6.58E-01 |
| rs1000954 | 4 | 110929483 | *CFI* | A/G | 0.28 | 0.19 | 6.59E-01 |
| rs17612 | 9 | 122765747 | *C5* | C/A | 0.07 | 0.19 | 6.61E-01 |
| rs11256448 | 10 | 6119485 | *IL2RA* | G/A | 0.25 | 0.19 | 6.63E-01 |
| rs17549193 | 9 | 136918847 | *FCN2* | T/C | 0.27 | 0.19 | 6.65E-01 |
| rs876650 | 3 | 188434334 | *MASP1* | C/G | 0.04 | 0.19 | 6.65E-01 |
| rs11720718 | 3 | 188424123 | *MASP1* | A/G | 0.41 | 0.18 | 6.72E-01 |
| rs12043615 | 1 | 205381011 | *C4BPA* | T/C | 0.22 | 0.17 | 6.79E-01 |
| rs2182913 | 1 | 205727001 | *CR2* | A/G | 0.31 | 0.17 | 6.79E-01 |
| rs4151657 | 6 | 32025519 | *CFB* | C/T | 0.33 | 0.17 | 6.83E-01 |
| rs17384711 | 5 | 39365431 | *C9* | C/T | 0.04 | 0.17 | 6.84E-01 |
| rs11120218 | 1 | 205345074 | *C4BPA* | A/G | 0.11 | 0.16 | 6.87E-01 |
| rs12139795 | 1 | 205697828 | *CR2* | G/A | 0.24 | 0.16 | 6.88E-01 |
| rs3774282 | 3 | 188464902 | *MASP1* | G/A | 0.32 | 0.16 | 6.91E-01 |
| rs11923275 | 3 | 188431367 | *MASP1* | G/A | 0.20 | 0.15 | 6.97E-01 |
| rs2749812 | 20 | 23010927 | *CD93* | A/G | 0.15 | 0.15 | 6.98E-01 |
| rs696405 | 3 | 188438917 | *MASP1* | A/C | 0.34 | 0.15 | 6.98E-01 |
| rs4926 | 11 | 57138565 | *SERPING1* | A/G | 0.30 | 0.15 | 6.98E-01 |
| rs3813729 | 12 | 7133345 | *CIR* | T/C | 0.08 | 0.15 | 7.01E-01 |
| rs1418471 | 1 | 57131889 | *C8A* | G/A | 0.16 | 0.14 | 7.05E-01 |
| rs624298 | 1 | 57147122 | *C8A* | T/C | 0.24 | 0.14 | 7.05E-01 |
| rs7713884 | 5 | 41000573 | *C7* | A/C | 0.20 | 0.14 | 7.06E-01 |
| rs11595876 | 10 | 54196768 | *MBL2* | C/T | 0.06 | 0.14 | 7.09E-01 |
| rs2416815 | 9 | 94472478 | intergenic | A/G | 0.06 | 0.13 | 7.18E-01 |
| rs11598648 | 10 | 6124031 | *IL2RA* | A/G | 0.42 | 0.13 | 7.20E-01 |
| rs3776526 | 5 | 39389479 | *C9* | G/A | 0.28 | 0.13 | 7.21E-01 |
| rs1063499 | 5 | 40991318 | *C7* | C/G | 0.41 | 0.13 | 7.21E-01 |
| rs7046 | 11 | 33683556 | *CD59* | T/C | 0.37 | 0.12 | 7.25E-01 |
| rs4315676 | 3 | 188429116 | *MASP1* | T/C | 0.04 | 0.12 | 7.27E-01 |
| rs11584921 | 1 | 205508598 | intergenic | G/A | 0.26 | 0.12 | 7.29E-01 |
| rs930507 | 10 | 54198272 | *MBL2* | G/C | 0.16 | 0.11 | 7.36E-01 |
| rs11672613 | 19 | 6656246 | *C3* | C/T | 0.42 | 0.11 | 7.39E-01 |
| rs3813946 | 1 | 205694316 | *CR2* | G/A | 0.24 | 0.11 | 7.42E-01 |
| rs1270942 | 6 | 32026839 | *CFB* | C/T | 0.12 | 0.10 | 7.49E-01 |
| rs2182911 | 1 | 205726694 | *CR2* | C/T | 0.20 | 0.10 | 7.53E-01 |
| rs428453 | 19 | 6653157 | *C3* | C/G | 0.40 | 0.10 | 7.54E-01 |
| rs1061443 | 5 | 41018379 | *C7* | T/G | 0.20 | 0.10 | 7.55E-01 |
| rs652785 | 1 | 57113315 | *C8A* | T/G | 0.39 | 0.10 | 7.55E-01 |
| rs669358 | 1 | 57177327 | *C8B* | G/A | 0.13 | 0.10 | 7.56E-01 |
| rs706781 | 10 | 6126391 | *IL2RA* | C/T | 0.27 | 0.09 | 7.62E-01 |
| rs17044525 | 1 | 205699419 | *CR2* | G/A | 0.10 | 0.09 | 7.65E-01 |
| rs2476491 | 10 | 6135416 | *IL2RA* | A/T | 0.27 | 0.09 | 7.68E-01 |
| rs7910961 | 10 | 6117802 | *IL2RA* | T/C | 0.32 | 0.09 | 7.70E-01 |
| rs2876849 | 5 | 41015547 | *C7* | T/A | 0.20 | 0.08 | 7.73E-01 |
| rs6671947 | 1 | 206003114 | *CD46* | G/C | 0.08 | 0.08 | 7.73E-01 |
| rs679350 | 1 | 57137400 | *C8A* | T/C | 0.27 | 0.08 | 7.79E-01 |
| rs4935346 | 10 | 54174739 | intergenic | A/T | 0.24 | 0.08 | 7.82E-01 |
| rs4749926 | 10 | 6125318 | *IL2RA* | A/G | 0.35 | 0.08 | 7.83E-01 |
| rs7527218 | 1 | 205690452 | intergenic | G/A | 0.48 | 0.07 | 7.91E-01 |
| rs3181268 | 11 | 33713749 | *CD59* | G/C | 0.21 | 0.07 | 7.92E-01 |
| rs6772329 | 3 | 188397297 | *RTP1* | T/C | 0.10 | 0.07 | 7.97E-01 |
| rs7072398 | 10 | 6119852 | *IL2RA* | G/A | 0.42 | 0.06 | 8.01E-01 |
| rs10494745 | 1 | 195154080 | *CFHR4* | A/G | 0.13 | 0.06 | 8.01E-01 |
| rs629409 | 1 | 22859325 | *C1QB* | A/G | 0.13 | 0.06 | 8.04E-01 |
| rs12722588 | 10 | 6100439 | *IL2RA* | A/G | 0.21 | 0.06 | 8.06E-01 |
| rs7093069 | 10 | 6103325 | *IL2RA* | T/C | 0.21 | 0.06 | 8.06E-01 |
| rs12685344 | 9 | 122804812 | *C5* | A/G | 0.11 | 0.06 | 8.08E-01 |
| rs7899538 | 10 | 6099904 | *IL2RA* | A/C | 0.11 | 0.06 | 8.09E-01 |
| rs2230199 | 19 | 6669387 | *C3* | G/C | 0.18 | 0.06 | 8.12E-01 |
| rs1833863 | 5 | 41125622 | intergenic | G/A | 0.27 | 0.06 | 8.14E-01 |
| rs3828511 | 5 | 41012092 | *C7* | T/G | 0.10 | 0.06 | 8.14E-01 |
| rs10513805 | 3 | 188433091 | *MASP1* | T/C | 0.05 | 0.05 | 8.18E-01 |
| rs1421094 | 5 | 39391348 | *C9* | A/G | 0.38 | 0.05 | 8.18E-01 |
| rs406514 | 19 | 6653598 | *C3* | C/T | 0.25 | 0.05 | 8.20E-01 |
| rs8112351 | 19 | 6657298 | *C3* | T/C | 0.25 | 0.05 | 8.20E-01 |
| rs885448 | 4 | 110910108 | *CFI* | A/T | 0.23 | 0.05 | 8.22E-01 |
| rs6669836 | 1 | 57178980 | *C8B* | C/T | 0.48 | 0.05 | 8.24E-01 |
| rs3805717 | 5 | 41197278 | *C6* | A/C | 0.15 | 0.05 | 8.27E-01 |
| rs4400166 | 5 | 41224048 | *C6* | G/A | 0.45 | 0.05 | 8.28E-01 |
| rs17201075 | 5 | 41221364 | *C6* | A/G | 0.07 | 0.04 | 8.38E-01 |
| rs16871040 | 5 | 41269943 | *C6* | T/A | 0.05 | 0.04 | 8.38E-01 |
| rs3805226 | 5 | 41009762 | *C7* | G/A | 0.10 | 0.04 | 8.39E-01 |
| rs550605 | 6 | 32015126 | *C2* | C/T | 0.09 | 0.04 | 8.40E-01 |
| rs3087554 | 8 | 27511359 | *CLU* | G/A | 0.17 | 0.04 | 8.41E-01 |
| rs656598 | 1 | 57182733 | *C8B* | T/G | 0.13 | 0.04 | 8.42E-01 |
| rs8942 | 1 | 205336542 | *C4BPB* | T/C | 0.15 | 0.04 | 8.49E-01 |
| rs6674522 | 1 | 195182982 | *CFHR2* | C/G | 0.14 | 0.03 | 8.53E-01 |
| rs7438961 | 4 | 110888100 | *CFI* | G/A | 0.31 | 0.03 | 8.55E-01 |
| rs1444903 | 5 | 41240060 | *C6* | T/C | 0.15 | 0.03 | 8.61E-01 |
| rs10941432 | 5 | 39395033 | *C9* | A/G | 0.47 | 0.03 | 8.63E-01 |
| rs12106739 | 3 | 188473505 | *MASP1* | G/A | 0.07 | 0.03 | 8.64E-01 |
| rs1801033 | 5 | 41235716 | *C6* | C/A | 0.37 | 0.03 | 8.69E-01 |
| rs1853881 | 1 | 195182368 | *CFHR2* | C/A | 0.16 | 0.03 | 8.69E-01 |
| rs534663 | 5 | 39376318 | *C9* | T/C | 0.43 | 0.03 | 8.70E-01 |
| rs17615 | 1 | 205713085 | *CR2* | A/G | 0.34 | 0.03 | 8.73E-01 |
| rs2298749 | 4 | 110900954 | *CFI* | A/G | 0.24 | 0.03 | 8.73E-01 |
| rs1801046 | 12 | 7133440 | *C1R* | T/C | 0.26 | 0.03 | 8.73E-01 |
| rs12489890 | 3 | 188435109 | *MASP1* | A/G | 0.15 | 0.02 | 8.78E-01 |
| rs831626 | 11 | 33702926 | *CD59* | G/A | 0.18 | 0.02 | 8.81E-01 |
| rs11671717 | 19 | 52500638 | intergenic | C/G | 0.23 | 0.02 | 8.82E-01 |
| rs17186771 | 1 | 205721816 | *CR2* | G/C | 0.25 | 0.02 | 8.84E-01 |
| rs547154 | 6 | 32018917 | *C2* | A/C | 0.09 | 0.02 | 8.86E-01 |
| rs541862 | 6 | 32024930 | *CFB* | G/A | 0.09 | 0.02 | 8.86E-01 |
| rs344550 | 19 | 6633953 | *C3* | G/C | 0.32 | 0.02 | 8.86E-01 |
| rs698084 | 3 | 188480342 | *MASP1* | A/G | 0.20 | 0.02 | 8.87E-01 |
| rs16840422 | 1 | 194919457 | *CFH* | T/C | 0.17 | 0.02 | 8.90E-01 |
| rs2241393 | 19 | 6636304 | *C3* | G/C | 0.38 | 0.02 | 8.93E-01 |
| rs850307 | 3 | 188464162 | *MASP1* | A/C | 0.24 | 0.02 | 8.93E-01 |
| rs1005511 | 11 | 57123232 | *SERPING1* | A/G | 0.30 | 0.02 | 8.97E-01 |
| rs9331888 | 8 | 27524779 | *CLU* | C/G | 0.26 | 0.02 | 9.00E-01 |
| rs1910016 | 5 | 40947349 | *C7* | C/T | 0.49 | 0.01 | 9.04E-01 |
| rs1738548 | 11 | 33708979 | *CD59* | C/T | 0.31 | 0.01 | 9.07E-01 |
| rs1838065 | 10 | 54199263 | *MBL2* | G/A | 0.36 | 0.01 | 9.07E-01 |
| rs698104 | 3 | 188485211 | *MASP1* | T/C | 0.14 | 0.01 | 9.12E-01 |
| rs17301153 | 1 | 57173572 | *C8B* | T/C | 0.16 | 0.01 | 9.12E-01 |
| rs2279590 | 8 | 27512170 | *CLU* | A/G | 0.42 | 0.01 | 9.17E-01 |
| rs10768024 | 11 | 33711053 | *CD59* | C/T | 0.11 | 0.01 | 9.24E-01 |
| rs12722563 | 10 | 6109567 | *IL2RA* | T/C | 0.12 | 0.01 | 9.31E-01 |
| rs3128623 | 9 | 136915930 | *FCN2* | C/T | 0.34 | 0.01 | 9.33E-01 |
| rs17514136 | 9 | 136912485 | *FCN2* | G/A | 0.26 | 0.01 | 9.34E-01 |
| rs6762462 | 3 | 188424749 | *MASP1* | A/C | 0.26 | 0.01 | 9.34E-01 |
| rs1838066 | 10 | 54199331 | *MBL2* | G/A | 0.36 | 0.01 | 9.35E-01 |
| rs1143679 | 16 | 31184312 | *CD11b* | A/G | 0.09 | 0.01 | 9.37E-01 |
| rs2256774 | 10 | 6137171 | *IL2RA* | G/A | 0.33 | 0.01 | 9.38E-01 |
| rs706779 | 10 | 6138830 | *IL2RA* | A/G | 0.47 | 0.01 | 9.38E-01 |
| rs4935047 | 10 | 54200073 | *MBL2* | A/G | 0.45 | 0.00 | 9.45E-01 |
| rs17216529 | 9 | 122840039 | *C5* | A/G | 0.07 | 0.00 | 9.46E-01 |
| rs4541508 | 4 | 110901936 | *CFI* | T/C | 0.33 | 0.00 | 9.51E-01 |
| rs2443040 | 5 | 40964331 | *C7* | G/T | 0.39 | 0.00 | 9.51E-01 |
| rs7037673 | 9 | 122780305 | *C5* | T/C | 0.39 | 0.00 | 9.51E-01 |
| rs6696924 | 1 | 57132516 | *C8A* | G/T | 0.27 | 0.00 | 9.52E-01 |
| rs2989726 | 9 | 136949195 | *FCN1* | G/A | 0.29 | 0.00 | 9.53E-01 |
| rs12085435 | 1 | 57187898 | *C8B* | A/G | 0.04 | 0.00 | 9.54E-01 |
| rs12067507 | 1 | 57195099 | *C8B* | T/C | 0.04 | 0.00 | 9.54E-01 |
| rs1546727 | 11 | 33682798 | *CD59* | A/G | 0.30 | 0.00 | 9.61E-01 |
| rs1013579 | 1 | 57195072 | *C8B* | G/A | 0.03 | 0.00 | 9.61E-01 |
| rs3733006 | 3 | 188453391 | *MASP1* | T/A | 0.12 | 0.00 | 9.65E-01 |
| rs641153 | 6 | 32022159 | *CFB* | T/C | 0.09 | 0.00 | 9.72E-01 |
| rs696764 | 5 | 39378250 | *C9* | A/G | 0.15 | 0.00 | 9.74E-01 |
| rs11585 | 11 | 33684281 | *CD59* | T/C | 0.08 | 0.00 | 9.75E-01 |
| rs3826945 | 19 | 813912 | *CFD* | G/A | 0.33 | 0.00 | 9.77E-01 |
| rs2192137 | 12 | 7045031 | *C1S* | A/G | 0.04 | 0.00 | 9.78E-01 |
| rs3746731 | 20 | 23013209 | *CD93* | G/A | 0.42 | 0.00 | 9.82E-01 |
| rs11117949 | 1 | 205748879 | *CR1* | C/T | 0.13 | 0.00 | 9.85E-01 |
| rs7545126 | 1 | 206004859 | *CD46* | T/C | 0.12 | 0.00 | 9.86E-01 |
| rs16861755 | 3 | 188429922 | *MASP1* | G/A | 0.04 | 0.00 | 9.86E-01 |
| rs7968584 | 12 | 7042502 | *C1S* | T/C | 0.21 | 0.00 | 9.88E-01 |
| rs16870514 | 5 | 40950922 | *C7* | A/G | 0.06 | 0.00 | 9.95E-01 |
| rs7900744 | 10 | 6105617 | *IL2RA* | G/A | 0.23 | 0.00 | 9.98E-01 |
| rs6540433 | 1 | 205720018 | *CR2* | C/A | 0.17 | 0.00 | 1 |

**Supplementary table 3**: Association results of SNPs genotyped over the complement genes within Paris cohort.

| **SNP** | **Ch** | **Position** | **Gene** | **Alleles** | **MAF** | **Chi-squared statistics** | **P-value** |
| --- | --- | --- | --- | --- | --- | --- | --- |
| rs2761434 | 1 | 205984122 | intergenic *CR1L-CD46* | A/G | 0.31 | 19.82 | 8.53E-06 |
| rs395544 | 1 | 194964895 | *CFH* | T/C | 0.34 | 19.22 | 1.16E-05 |
| rs7416336 | 1 | 195138798 | *CFHR4* | G/C | 0.27 | 19.20 | 1.17E-05 |
| rs1065489 | 1 | 194976397 | *CFH* | T/G | 0.28 | 19.17 | 1.19E-05 |
| rs1329423 | 1 | 194913010 | *CFH* | G/A | 0.36 | 19.08 | 1.25E-05 |
| rs3795341 | 1 | 195153897 | *CFHR4* | T/C | 0.28 | 18.71 | 1.52E-05 |
| rs12405238 | 1 | 194928236 | *CFH* | T/G | 0.36 | 17.97 | 2.24E-05 |
| rs3753396 | 1 | 194962365 | *CFH* | G/A | 0.27 | 17.62 | 2.70E-05 |
| rs1061170 | 1 | 194925860 | *CFH* | C/T | 0.30 | 17.42 | 3.00E-05 |
| rs10922102 | 1 | 194934910 | *CFH* | C/T | 0.30 | 17.12 | 3.50E-05 |
| rs7529589 | 1 | 194924902 | *CFH* | T/C | 0.31 | 16.74 | 4.28E-05 |
| rs424535 | 1 | 194975846 | *CFH* | A/T | 0.49 | 16.65 | 4.50E-05 |
| rs9427934 | 1 | 195189483 | *CFHR2* | A/G | 0.43 | 16.25 | 5.54E-05 |
| rs10922096 | 1 | 194929082 | *CFH* | C/T | 0.48 | 16.13 | 5.93E-05 |
| rs514943 | 1 | 194930536 | *CFH* | G/A | 0.30 | 15.51 | 8.19E-05 |
| rs10449303 | 1 | 206027681 | *CD46* | G/A | 0.43 | 14.76 | 1.22E-04 |
| rs6685931 | 1 | 195133856 | *CFHR4* | C/T | 0.32 | 14.30 | 1.56E-04 |
| rs1853883 | 1 | 195148223 | *CFHR4* | C/G | 0.37 | 13.83 | 2.00E-04 |
| rs2796275 | 1 | 206017113 | *CD46* | C/T | 0.39 | 13.75 | 2.08E-04 |
| rs6695321 | 1 | 194942484 | *CFH* | G/A | 0.44 | 13.53 | 2.35E-04 |
| rs2796278 | 1 | 206022446 | *CD46* | C/A | 0.46 | 12.07 | 5.11E-04 |
| rs7144 | 1 | 206034342 | *CD46* | C/T | 0.45 | 12.07 | 5.13E-04 |
| rs1652333 | 1 | 205537083 | intergenic *C4BPA-CR1* | C/T | 0.29 | 9.98 | 1.58E-03 |
| rs12032512 | 1 | 205719801 | *CR2* | G/C | 0.43 | 9.81 | 1.74E-03 |
| rs1971579 | 1 | 195153804 | *CFHR4* | C/A | 0.37 | 8.69 | 3.20E-03 |
| rs2071006 | 9 | 138959725 | *C8G* | T/G | 0.40 | 6.81 | 9.06E-03 |
| rs17040805 | 4 | 110884246 | *CFI* | A/C | 0.07 | 6.56 | 1.05E-02 |
| rs4844573 | 1 | 205371523 | *C4BPA* | C/T | 0.39 | 6.17 | 1.30E-02 |
| rs12043615 | 1 | 205381011 | *C4BPA* | T/C | 0.19 | 5.81 | 1.60E-02 |
| rs12722596 | 10 | 6096300 | *IL2RA* | G/A | 0.09 | 5.79 | 1.61E-02 |
| rs16870514 | 5 | 40950922 | *C7* | A/G | 0.09 | 5.14 | 2.34E-02 |
| rs6661764 | 1 | 205875008 | *CR1* | G/C | 0.28 | 4.59 | 3.21E-02 |
| rs2039544 | 1 | 205486540 | intergenic | T/G | 0.48 | 4.55 | 3.29E-02 |
| rs2491393 | 1 | 205366882 | *C4BPA* | G/A | 0.38 | 4.46 | 3.46E-02 |
| rs3748557 | 1 | 195213492 | *CFHR5* | T/A | 0.27 | 4.22 | 4.00E-02 |
| rs7533852 | 1 | 205562050 | *CD55* | T/G | 0.48 | 4.08 | 4.34E-02 |
| rs1800450 | 10 | 54201241 | *MBL2* | A/G | 0.12 | 3.84 | 4.99E-02 |
| rs2256774 | 10 | 6137171 | *IL2RA* | G/A | 0.45 | 3.75 | 5.27E-02 |
| rs3805717 | 5 | 41197278 | *C6* | A/C | 0.16 | 3.71 | 5.40E-02 |
| rs7555070 | 1 | 195248777 | intergenic *CFHR5-F13B* | C/T | 0.40 | 3.67 | 5.55E-02 |
| rs10127904 | 1 | 205814730 | *CR1* | G/T | 0.29 | 3.64 | 5.65E-02 |
| rs4151667 | 6 | 32022003 | *CFB* | A/T | 0.03 | 3.61 | 5.73E-02 |
| rs1126618 | 1 | 205364303 | *C4BPA* | T/C | 0.16 | 3.59 | 5.83E-02 |
| rs12614 | 6 | 32022158 | *CFB* | T/C | 0.14 | 3.56 | 5.92E-02 |
| rs419137 | 1 | 194963498 | *CFH* | C/A | 0.11 | 3.48 | 6.21E-02 |
| rs16871040 | 5 | 41269943 | *C6* | T/A | 0.06 | 3.36 | 6.69E-02 |
| rs17687664 | 11 | 33696988 | *CD59* | C/G | 0.12 | 3.28 | 7.01E-02 |
| rs698092 | 3 | 188452328 | *MASP1* | G/A | 0.41 | 3.23 | 7.22E-02 |
| rs1047286 | 19 | 6664262 | *C3* | T/C | 0.19 | 3.20 | 7.36E-02 |
| rs2084624 | 5 | 40986565 | *C7* | C/T | 0.21 | 3.13 | 7.70E-02 |
| rs17044525 | 1 | 205699419 | *CR2* | G/A | 0.09 | 3.11 | 7.77E-02 |
| rs2182911 | 1 | 205726694 | *CR2* | C/T | 0.17 | 3.11 | 7.78E-02 |
| rs831627 | 11 | 33702623 | *CD59* | T/C | 0.03 | 3.11 | 7.80E-02 |
| rs9332739 | 6 | 32011783 | *C2* | C/G | 0.04 | 3.06 | 8.03E-02 |
| rs2796269 | 1 | 206003509 | *CD46* | T/C | 0.29 | 3.06 | 8.05E-02 |
| rs11569546 | 19 | 6634749 | *C3* | T/C | 0.11 | 3.05 | 8.06E-02 |
| rs6875226 | 5 | 40979199 | *C7* | C/T | 0.22 | 3.04 | 8.13E-02 |
| rs2273121 | 11 | 33714346 | *CD59* | T/C | 0.21 | 2.95 | 8.58E-02 |
| rs3776526 | 5 | 39389479 | *C9* | G/A | 0.22 | 2.92 | 8.72E-02 |
| rs10473238 | 5 | 41231987 | *C6* | T/C | 0.08 | 2.87 | 9.02E-02 |
| rs6656401 | 1 | 205758672 | *CR1* | A/G | 0.16 | 2.77 | 9.58E-02 |
| rs823940 | 3 | 188462698 | *MASP1* | G/A | 0.12 | 2.74 | 9.76E-02 |
| rs6860770 | 5 | 41221669 | *C6* | C/A | 0.07 | 2.64 | 1.04E-01 |
| rs324056 | 5 | 40968997 | *C7* | T/G | 0.19 | 2.62 | 1.05E-01 |
| rs10922153 | 1 | 195245238 | *CFHR5* | G/T | 0.48 | 2.57 | 1.09E-01 |
| rs2455313 | 5 | 40982362 | *C7* | A/T | 0.23 | 2.56 | 1.10E-01 |
| rs2274567 | 1 | 205820244 | *CR1* | G/A | 0.18 | 2.50 | 1.13E-01 |
| rs2675982 | 5 | 40996035 | *C7* | A/G | 0.06 | 2.48 | 1.15E-01 |
| rs17615 | 1 | 205713085 | *CR2* | A/G | 0.29 | 2.43 | 1.19E-01 |
| rs7519408 | 1 | 205727912 | *CR2* | G/C | 0.11 | 2.42 | 1.20E-01 |
| rs10494745 | 1 | 195154080 | *CFHR4* | A/G | 0.07 | 2.37 | 1.24E-01 |
| rs12740591 | 1 | 22837590 | *C1QA* | T/C | 0.04 | 2.34 | 1.26E-01 |
| rs3746732 | 20 | 23013342 | *CD93* | A/G | 0.23 | 2.30 | 1.29E-01 |
| rs12722486 | 10 | 6143768 | *IL2RA* | A/G | 0.06 | 2.25 | 1.34E-01 |
| rs2230199 | 19 | 6669387 | *C3* | G/C | 0.21 | 2.25 | 1.34E-01 |
| rs8048583 | 16 | 31187037 | *CD11B* | T/C | 0.30 | 2.23 | 1.36E-01 |
| rs11959122 | 5 | 39346359 | *C9* | T/C | 0.12 | 2.18 | 1.40E-01 |
| rs1013579 | 1 | 57195072 | *C8B* | G/A | 0.03 | 2.18 | 1.40E-01 |
| rs698093 | 3 | 188452845 | *MASP1* | T/G | 0.27 | 2.15 | 1.42E-01 |
| rs3746731 | 20 | 23013209 | *CD93* | G/A | 0.44 | 2.14 | 1.43E-01 |
| rs2296160 | 1 | 205861943 | *CR1* | T/C | 0.19 | 2.12 | 1.45E-01 |
| rs11117949 | 1 | 205748879 | *CR1* | C/T | 0.18 | 2.01 | 1.57E-01 |
| rs1389623 | 19 | 6635197 | *C3* | T/C | 0.13 | 1.99 | 1.59E-01 |
| rs4315676 | 3 | 188429116 | *MASP1* | T/C | 0.07 | 1.94 | 1.64E-01 |
| rs17259045 | 1 | 205849330 | *CR1* | G/A | 0.12 | 1.92 | 1.65E-01 |
| rs10512764 | 5 | 41193097 | *C6* | T/C | 0.12 | 1.86 | 1.73E-01 |
| rs1838065 | 10 | 54199263 | *MBL2* | G/A | 0.38 | 1.80 | 1.80E-01 |
| rs17186575 | 1 | 205351774 | *C4BPA* | A/G | 0.06 | 1.80 | 1.80E-01 |
| rs17468519 | 5 | 39339216 | *C9* | A/G | 0.07 | 1.79 | 1.81E-01 |
| rs3774279 | 3 | 188463795 | *MASP1* | G/A | 0.29 | 1.78 | 1.82E-01 |
| rs4597342 | 16 | 31251270 | *CD11B* | T/C | 0.27 | 1.77 | 1.84E-01 |
| rs1546727 | 11 | 33682798 | *CD59* | A/G | 0.30 | 1.73 | 1.88E-01 |
| rs4926 | 11 | 57138565 | *SERPING1* | A/G | 0.28 | 1.69 | 1.94E-01 |
| rs6683663 | 1 | 57129064 | *C8A* | G/A | 0.19 | 1.67 | 1.97E-01 |
| rs7183 | 12 | 7048280 | *C1S* | T/G | 0.11 | 1.67 | 1.97E-01 |
| rs2230204 | 19 | 6660848 | *C3* | T/C | 0.30 | 1.67 | 1.97E-01 |
| rs11951782 | 5 | 41227310 | *C6* | A/T | 0.13 | 1.65 | 2.00E-01 |
| rs1853881 | 1 | 195182368 | *CFHR2* | C/A | 0.10 | 1.64 | 2.01E-01 |
| rs12734030 | 1 | 205860587 | *CR1* | T/C | 0.17 | 1.62 | 2.03E-01 |
| rs1421094 | 5 | 39391348 | *C9* | A/G | 0.43 | 1.60 | 2.06E-01 |
| rs1838066 | 10 | 54199331 | *MBL2* | G/A | 0.38 | 1.56 | 2.11E-01 |
| rs9429945 | 1 | 205743391 | *CR1* | T/C | 0.17 | 1.53 | 2.15E-01 |
| rs6697228 | 1 | 57122052 | *C8A* | C/T | 0.14 | 1.52 | 2.18E-01 |
| rs4541508 | 4 | 110901936 | *CFI* | T/C | 0.37 | 1.49 | 2.23E-01 |
| rs13089330 | 3 | 188492964 | *MASP1* | T/C | 0.27 | 1.47 | 2.25E-01 |
| rs2274037 | 10 | 6102114 | *IL2RA* | A/G | 0.04 | 1.43 | 2.31E-01 |
| rs6694643 | 1 | 57121995 | *C8A* | T/A | 0.14 | 1.42 | 2.33E-01 |
| rs6665881 | 1 | 205723228 | *CR2* | C/G | 0.42 | 1.36 | 2.43E-01 |
| rs11032349 | 11 | 33686248 | *CD59* | T/A | 0.11 | 1.35 | 2.44E-01 |
| rs3864098 | 3 | 188478554 | *MASP1* | C/T | 0.21 | 1.35 | 2.45E-01 |
| rs324062 | 5 | 40956290 | *C7* | A/C | 0.26 | 1.35 | 2.45E-01 |
| rs14374 | 1 | 206034880 | *CD46* | C/T | 0.03 | 1.34 | 2.47E-01 |
| rs17030 | 19 | 6628989 | *C3* | G/A | 0.50 | 1.33 | 2.49E-01 |
| rs2025345 | 10 | 6107694 | *IL2RA* | G/A | 0.41 | 1.31 | 2.52E-01 |
| rs11136000 | 8 | 27520436 | *CLU* | T/C | 0.44 | 1.30 | 2.55E-01 |
| rs11598648 | 10 | 6124031 | *IL2RA* | A/G | 0.41 | 1.29 | 2.56E-01 |
| rs4077810 | 16 | 31248410 | *CD11B* | T/C | 0.28 | 1.29 | 2.57E-01 |
| rs7951 | 19 | 6632991 | *C3* | T/C | 0.09 | 1.27 | 2.59E-01 |
| rs13064994 | 3 | 188488710 | *MASP1* | A/G | 0.22 | 1.27 | 2.60E-01 |
| rs11740241 | 5 | 39387223 | *C9* | C/T | 0.33 | 1.27 | 2.60E-01 |
| rs2230203 | 19 | 6661782 | *C3* | A/C | 0.16 | 1.27 | 2.60E-01 |
| rs1005511 | 11 | 57123232 | *SERPING1* | A/G | 0.33 | 1.25 | 2.64E-01 |
| rs17044576 | 1 | 205701293 | *CR2* | C/T | 0.08 | 1.24 | 2.66E-01 |
| rs1738548 | 11 | 33708979 | *CD59* | C/T | 0.34 | 1.23 | 2.67E-01 |
| rs1877134 | 10 | 54184769 | intergenic | G/A | 0.43 | 1.20 | 2.74E-01 |
| rs17384711 | 5 | 39365431 | *C9* | C/T | 0.03 | 1.18 | 2.77E-01 |
| rs2191959 | 9 | 122723655 | *TRAF1* | A/T | 0.07 | 1.16 | 2.82E-01 |
| rs1887632 | 1 | 205738244 | *CR1* | A/G | 0.07 | 1.16 | 2.82E-01 |
| rs3181268 | 11 | 33713749 | *CD59* | G/C | 0.19 | 1.12 | 2.90E-01 |
| rs3737002 | 1 | 205827396 | *CR1* | T/C | 0.27 | 1.11 | 2.91E-01 |
| rs1143683 | 16 | 31244389 | *CD11B* | T/C | 0.19 | 1.11 | 2.92E-01 |
| rs656598 | 1 | 57182733 | *C8B* | T/G | 0.13 | 1.11 | 2.92E-01 |
| rs696764 | 5 | 39378250 | *C9* | A/G | 0.18 | 1.11 | 2.93E-01 |
| rs4749926 | 10 | 6125318 | *IL2RA* | A/G | 0.44 | 1.09 | 2.96E-01 |
| rs11206932 | 1 | 57134595 | *C8A* | A/T | 0.13 | 1.09 | 2.96E-01 |
| rs1048709 | 6 | 32022914 | *CFB* | A/G | 0.13 | 1.08 | 2.98E-01 |
| rs1774900 | 1 | 57149737 | *C8A* | C/G | 0.10 | 1.06 | 3.04E-01 |
| rs2749813 | 20 | 23010984 | *CD93* | T/C | 0.31 | 1.05 | 3.06E-01 |
| rs1571344 | 1 | 205737551 | *CR1* | C/T | 0.11 | 1.03 | 3.10E-01 |
| rs294185 | 1 | 22844479 | *C1QC* | C/T | 0.42 | 1.03 | 3.11E-01 |
| rs698090 | 3 | 188446994 | *MASP1* | G/A | 0.38 | 1.02 | 3.12E-01 |
| rs4571969 | 1 | 205372211 | *C4BPA* | T/C | 0.28 | 1.02 | 3.12E-01 |
| rs867231 | 8 | 27523309 | *CLU* | G/C | 0.23 | 1.01 | 3.14E-01 |
| rs17760306 | 11 | 33694612 | *CD59* | G/T | 0.15 | 1.00 | 3.17E-01 |
| rs2277984 | 19 | 6630511 | *C3* | G/A | 0.50 | 0.98 | 3.21E-01 |
| rs1143678 | 16 | 31250506 | *CD11B* | T/C | 0.18 | 0.98 | 3.23E-01 |
| rs2300955 | 1 | 57116750 | *C8A* | T/C | 0.17 | 0.96 | 3.28E-01 |
| rs4698788 | 4 | 110938983 | *CFI* | C/T | 0.06 | 0.95 | 3.29E-01 |
| rs11721403 | 4 | 110913018 | *CFI* | G/A | 0.42 | 0.93 | 3.34E-01 |
| rs11645653 | 16 | 31220356 | *CD11B* | C/T | 0.23 | 0.91 | 3.39E-01 |
| rs1143679 | 16 | 31184312 | *CD11B* | A/G | 0.14 | 0.91 | 3.40E-01 |
| rs291990 | 1 | 22852242 | *C1QB* | C/A | 0.49 | 0.91 | 3.40E-01 |
| rs10794501 | 1 | 27572036 | *FCN3* | A/T | 0.29 | 0.90 | 3.43E-01 |
| rs17258982 | 1 | 205719987 | *CR2* | G/A | 0.08 | 0.89 | 3.45E-01 |
| rs10513805 | 3 | 188433091 | *MASP1* | T/C | 0.06 | 0.88 | 3.47E-01 |
| rs7910961 | 10 | 6117802 | *IL2RA* | T/C | 0.39 | 0.87 | 3.51E-01 |
| rs1831150 | 1 | 205737061 | *CR1* | G/A | 0.27 | 0.86 | 3.53E-01 |
| rs652785 | 1 | 57113315 | *C8A* | T/G | 0.36 | 0.86 | 3.54E-01 |
| rs16861755 | 3 | 188429922 | *MASP1* | G/A | 0.06 | 0.86 | 3.55E-01 |
| rs3774268 | 3 | 188437018 | *MASP1* | A/G | 0.15 | 0.84 | 3.58E-01 |
| rs7545083 | 1 | 205362511 | *C4BPA* | C/T | 0.35 | 0.83 | 3.61E-01 |
| rs1910016 | 5 | 40947349 | *C7* | C/T | 0.38 | 0.83 | 3.63E-01 |
| rs11064497 | 12 | 7039922 | *C1S* | T/C | 0.12 | 0.82 | 3.65E-01 |
| rs7438961 | 4 | 110888100 | *CFI* | G/A | 0.34 | 0.82 | 3.66E-01 |
| rs6671947 | 1 | 206003114 | *CD46* | G/C | 0.08 | 0.79 | 3.73E-01 |
| rs831628 | 11 | 33701421 | *CD59* | G/C | 0.19 | 0.79 | 3.75E-01 |
| rs599857 | 1 | 57201177 | *C8B* | C/T | 0.27 | 0.78 | 3.78E-01 |
| rs2025935 | 1 | 205742278 | *CR1* | T/C | 0.27 | 0.78 | 3.78E-01 |
| rs9200 | 5 | 41178363 | *C6* | A/G | 0.48 | 0.75 | 3.86E-01 |
| rs7193268 | 16 | 31248498 | *CD11B* | T/C | 0.19 | 0.75 | 3.88E-01 |
| rs6565228 | 16 | 31236781 | *CD11B* | A/G | 0.18 | 0.74 | 3.90E-01 |
| rs292001 | 1 | 22837545 | *C1QA* | A/G | 0.45 | 0.74 | 3.91E-01 |
| rs6669836 | 1 | 57178980 | *C8B* | C/T | 0.48 | 0.72 | 3.98E-01 |
| rs12722588 | 10 | 6100439 | *IL2RA* | A/G | 0.17 | 0.71 | 4.00E-01 |
| rs7093069 | 10 | 6103325 | *IL2RA* | T/C | 0.17 | 0.71 | 4.00E-01 |
| rs706781 | 10 | 6126391 | *IL2RA* | C/T | 0.25 | 0.70 | 4.03E-01 |
| rs1270942 | 6 | 32026839 | *CFB* | C/T | 0.08 | 0.70 | 4.03E-01 |
| rs2182913 | 1 | 205727001 | *CR2* | A/G | 0.28 | 0.70 | 4.03E-01 |
| rs1001073 | 3 | 188427399 | *MASP1* | G/A | 0.29 | 0.70 | 4.03E-01 |
| rs867230 | 8 | 27524420 | *CLU* | C/A | 0.43 | 0.69 | 4.06E-01 |
| rs9331930 | 8 | 27514211 | *CLU* | C/A | 0.26 | 0.68 | 4.10E-01 |
| rs7545126 | 1 | 206004859 | *CD46* | T/C | 0.09 | 0.68 | 4.10E-01 |
| rs2989725 | 9 | 136947826 | *FCN1* | C/A | 0.35 | 0.68 | 4.10E-01 |
| rs17301153 | 1 | 57173572 | *C8B* | T/C | 0.19 | 0.66 | 4.18E-01 |
| rs9937837 | 16 | 31206440 | *CD11B* | G/T | 0.32 | 0.65 | 4.21E-01 |
| rs3828511 | 5 | 41012092 | *C7* | T/G | 0.11 | 0.64 | 4.22E-01 |
| rs2842704 | 1 | 205356059 | *C4BPA* | G/A | 0.17 | 0.64 | 4.23E-01 |
| rs9888879 | 16 | 31217873 | *CD11B* | C/T | 0.18 | 0.64 | 4.24E-01 |
| rs2842707 | 1 | 205364107 | *C4BPA* | C/G | 0.46 | 0.64 | 4.25E-01 |
| rs706778 | 10 | 6138955 | *IL2RA* | A/G | 0.37 | 0.64 | 4.25E-01 |
| rs641153 | 6 | 32022159 | *CFB* | T/C | 0.14 | 0.62 | 4.32E-01 |
| rs2301247 | 5 | 41190285 | *C6* | G/A | 0.39 | 0.62 | 4.32E-01 |
| rs10941432 | 5 | 39395033 | *C9* | A/G | 0.42 | 0.62 | 4.33E-01 |
| rs7037673 | 9 | 122780305 | *C5* | T/C | 0.41 | 0.61 | 4.34E-01 |
| rs710473 | 3 | 188475684 | *MASP1* | G/A | 0.23 | 0.61 | 4.34E-01 |
| rs3749519 | 5 | 41017132 | *C7* | G/A | 0.35 | 0.61 | 4.34E-01 |
| rs2192137 | 12 | 7045031 | *C1S* | A/G | 0.04 | 0.60 | 4.38E-01 |
| rs7900744 | 10 | 6105617 | *IL2RA* | G/A | 0.21 | 0.60 | 4.38E-01 |
| rs11585 | 11 | 33684281 | *CD59* | T/C | 0.08 | 0.60 | 4.40E-01 |
| rs10818494 | 9 | 122786259 | *C5* | C/T | 0.41 | 0.59 | 4.43E-01 |
| rs9663421 | 10 | 6095610 | *IL2RA* | T/C | 0.31 | 0.59 | 4.43E-01 |
| rs2476491 | 10 | 6135416 | *IL2RA* | A/T | 0.37 | 0.58 | 4.47E-01 |
| rs7308467 | 12 | 7134924 | *CIR* | C/T | 0.29 | 0.58 | 4.48E-01 |
| rs3774282 | 3 | 188464902 | *MASP1* | G/A | 0.40 | 0.58 | 4.48E-01 |
| rs11671717 | 19 | 52500638 | intergenic | C/G | 0.26 | 0.57 | 4.49E-01 |
| rs11569429 | 19 | 6660074 | *C3* | A/G | 0.14 | 0.57 | 4.49E-01 |
| rs1000954 | 4 | 110929483 | *CFI* | A/G | 0.28 | 0.56 | 4.56E-01 |
| rs1423394 | 5 | 41152600 | intergenic | C/T | 0.28 | 0.56 | 4.56E-01 |
| rs11596355 | 10 | 6104187 | *IL2RA* | C/T | 0.07 | 0.55 | 4.57E-01 |
| rs7040033 | 9 | 122798865 | *C5* | A/G | 0.45 | 0.55 | 4.58E-01 |
| rs12085435 | 1 | 57187898 | *C8B* | A/G | 0.05 | 0.55 | 4.59E-01 |
| rs12067507 | 1 | 57195099 | *C8B* | T/C | 0.05 | 0.55 | 4.59E-01 |
| rs614709 | 1 | 205819898 | *CR1* | C/T | 0.23 | 0.55 | 4.60E-01 |
| rs6691117 | 1 | 205849554 | *CR1* | G/A | 0.23 | 0.55 | 4.60E-01 |
| rs3107217 | 3 | 188479513 | *MASP1* | T/A | 0.08 | 0.54 | 4.61E-01 |
| rs10905668 | 10 | 6132061 | *IL2RA* | T/C | 0.20 | 0.53 | 4.65E-01 |
| rs3774275 | 3 | 188447962 | *MASP1* | G/A | 0.27 | 0.53 | 4.65E-01 |
| rs17611 | 9 | 122809021 | *C5* | A/G | 0.44 | 0.53 | 4.67E-01 |
| rs25681 | 9 | 122819826 | *C5* | T/C | 0.44 | 0.53 | 4.67E-01 |
| rs12116668 | 1 | 57143721 | *C8A* | C/T | 0.12 | 0.52 | 4.73E-01 |
| rs3813729 | 12 | 7133345 | *CIR* | T/C | 0.12 | 0.51 | 4.73E-01 |
| rs11957341 | 5 | 41233229 | *C6* | A/C | 0.40 | 0.51 | 4.76E-01 |
| rs12711521 | 1 | 11013503 | *MASP2* | C/A | 0.23 | 0.50 | 4.79E-01 |
| rs700233 | 5 | 39400311 | *C9* | A/G | 0.45 | 0.50 | 4.80E-01 |
| rs1063499 | 5 | 40991318 | *C7* | C/G | 0.47 | 0.49 | 4.86E-01 |
| rs1105323 | 9 | 136942234 | *FCN1* | A/G | 0.40 | 0.47 | 4.93E-01 |
| rs550605 | 6 | 32015126 | *C2* | C/T | 0.14 | 0.47 | 4.94E-01 |
| rs547154 | 6 | 32018917 | *C2* | A/C | 0.14 | 0.47 | 4.94E-01 |
| rs541862 | 6 | 32024930 | *CFB* | G/A | 0.14 | 0.47 | 4.94E-01 |
| rs16910280 | 9 | 122844597 | *C5* | T/C | 0.18 | 0.47 | 4.95E-01 |
| rs6848178 | 4 | 110885620 | *CFI* | T/A | 0.46 | 0.45 | 5.04E-01 |
| rs7499077 | 16 | 31225006 | *CD11B* | A/G | 0.32 | 0.44 | 5.06E-01 |
| rs10818495 | 9 | 122797008 | *C5* | A/C | 0.48 | 0.44 | 5.06E-01 |
| rs850312 | 3 | 188436502 | *MASP1* | A/G | 0.30 | 0.44 | 5.07E-01 |
| rs1474891 | 1 | 11004467 | *TARDBP* | C/A | 0.23 | 0.44 | 5.09E-01 |
| rs155377 | 5 | 39344808 | *C9* | C/T | 0.46 | 0.43 | 5.10E-01 |
| rs3843010 | 3 | 188419741 | *MASP1* | T/G | 0.38 | 0.43 | 5.11E-01 |
| rs292010 | 1 | 22862332 | intergenic | A/G | 0.08 | 0.42 | 5.16E-01 |
| rs7732104 | 5 | 41019193 | *C7* | A/G | 0.15 | 0.42 | 5.17E-01 |
| rs3805226 | 5 | 41009762 | *C7* | G/A | 0.12 | 0.42 | 5.17E-01 |
| rs1423396 | 5 | 41158467 | intergenic | G/A | 0.21 | 0.41 | 5.20E-01 |
| rs7713884 | 5 | 41000573 | *C7* | A/C | 0.25 | 0.41 | 5.21E-01 |
| rs4400166 | 5 | 41224048 | *C6* | G/A | 0.48 | 0.41 | 5.22E-01 |
| rs9331888 | 8 | 27524779 | *CLU* | C/G | 0.27 | 0.41 | 5.22E-01 |
| rs624298 | 1 | 57147122 | *C8A* | T/C | 0.24 | 0.41 | 5.23E-01 |
| rs7851696 | 9 | 136918912 | *FCN2* | T/G | 0.12 | 0.41 | 5.24E-01 |
| rs16861893 | 3 | 188491870 | *MASP1* | A/G | 0.07 | 0.40 | 5.25E-01 |
| rs800292 | 1 | 194908856 | *CFH* | T/C | 0.21 | 0.40 | 5.26E-01 |
| rs2443040 | 5 | 40964331 | *C7* | G/T | 0.46 | 0.40 | 5.27E-01 |
| rs7492 | 20 | 23008257 | *CD93* | A/G | 0.08 | 0.39 | 5.31E-01 |
| rs3128626 | 9 | 136913377 | *FCN2* | T/C | 0.30 | 0.39 | 5.33E-01 |
| rs1801046 | 12 | 7133440 | *C1R* | T/C | 0.26 | 0.38 | 5.36E-01 |
| rs17040 | 3 | 188420262 | *MASP1* | T/C | 0.45 | 0.38 | 5.36E-01 |
| rs11150610 | 16 | 31241737 | *CD11B* | A/C | 0.39 | 0.38 | 5.36E-01 |
| rs10768024 | 11 | 33711053 | *CD59* | C/T | 0.12 | 0.38 | 5.40E-01 |
| rs696405 | 3 | 188438917 | *MASP1* | A/C | 0.39 | 0.37 | 5.42E-01 |
| rs11953839 | 5 | 39365195 | *C9* | A/G | 0.42 | 0.36 | 5.48E-01 |
| rs669358 | 1 | 57177327 | *C8B* | G/A | 0.13 | 0.35 | 5.53E-01 |
| rs9880761 | 3 | 188423991 | *MASP1* | G/A | 0.32 | 0.35 | 5.54E-01 |
| rs7671905 | 4 | 110942335 | *CFI* | T/C | 0.31 | 0.34 | 5.58E-01 |
| rs12021671 | 1 | 205724768 | *CR2* | A/G | 0.38 | 0.34 | 5.59E-01 |
| rs791587 | 10 | 6128705 | *IL2RA* | A/G | 0.45 | 0.34 | 5.61E-01 |
| rs7026551 | 9 | 122772954 | *C5* | C/A | 0.19 | 0.33 | 5.63E-01 |
| rs874603 | 3 | 188435731 | *MASP1* | G/A | 0.11 | 0.33 | 5.66E-01 |
| rs7954916 | 12 | 8109659 | *C3AR1* | A/C | 0.27 | 0.33 | 5.68E-01 |
| rs11256433 | 10 | 6117851 | *IL2RA* | G/T | 0.19 | 0.32 | 5.73E-01 |
| rs2241392 | 19 | 6636983 | *C3* | G/C | 0.35 | 0.31 | 5.75E-01 |
| rs704697 | 11 | 33681356 | *CD59* | T/C | 0.43 | 0.31 | 5.76E-01 |
| rs1035497 | 5 | 41205972 | *C6* | G/A | 0.14 | 0.31 | 5.77E-01 |
| rs3181274 | 11 | 33711865 | *CD59* | A/G | 0.38 | 0.31 | 5.77E-01 |
| rs4522435 | 16 | 31269424 | intergenic | T/C | 0.07 | 0.31 | 5.79E-01 |
| rs4915309 | 1 | 170174251 | intergenic | A/T | 0.13 | 0.30 | 5.84E-01 |
| rs7746553 | 6 | 32003952 | *C2* | G/C | 0.14 | 0.30 | 5.86E-01 |
| rs3733006 | 3 | 188453391 | *MASP1* | T/A | 0.13 | 0.29 | 5.91E-01 |
| rs696760 | 5 | 39369251 | *C9* | C/G | 0.10 | 0.29 | 5.91E-01 |
| rs432823 | 19 | 6653246 | *C3* | A/G | 0.38 | 0.29 | 5.92E-01 |
| rs658285 | 1 | 57188923 | *C8B* | T/C | 0.31 | 0.29 | 5.92E-01 |
| rs831636 | 11 | 33696174 | *CD59* | T/C | 0.29 | 0.29 | 5.92E-01 |
| rs11672613 | 19 | 6656246 | *C3* | C/T | 0.41 | 0.28 | 5.96E-01 |
| rs12087872 | 1 | 205373156 | *C4BPA* | G/A | 0.26 | 0.28 | 5.98E-01 |
| rs11923275 | 3 | 188431367 | *MASP1* | G/A | 0.21 | 0.28 | 5.99E-01 |
| rs3805713 | 5 | 41189118 | *C6* | A/T | 0.36 | 0.27 | 6.01E-01 |
| rs2269066 | 9 | 122776839 | *C5* | T/C | 0.10 | 0.27 | 6.06E-01 |
| rs1048971 | 1 | 205712945 | *CR2* | A/G | 0.39 | 0.26 | 6.08E-01 |
| rs428453 | 19 | 6653157 | *C3* | C/G | 0.38 | 0.26 | 6.09E-01 |
| rs7257062 | 19 | 6636945 | *C3* | T/C | 0.42 | 0.26 | 6.11E-01 |
| rs6822976 | 4 | 110885741 | *CFI* | A/G | 0.46 | 0.26 | 6.11E-01 |
| rs17186771 | 1 | 205721816 | *CR2* | G/C | 0.22 | 0.25 | 6.14E-01 |
| rs918608 | 5 | 41188446 | *C6* | C/T | 0.18 | 0.25 | 6.14E-01 |
| rs835209 | 5 | 39386120 | *C9* | T/C | 0.03 | 0.25 | 6.16E-01 |
| rs6602398 | 10 | 6122959 | *IL2RA* | T/G | 0.35 | 0.25 | 6.19E-01 |
| rs2386841 | 10 | 6097738 | *IL2RA* | A/C | 0.16 | 0.24 | 6.25E-01 |
| rs11584921 | 1 | 205508598 | intergenic | G/A | 0.21 | 0.24 | 6.27E-01 |
| rs16910255 | 9 | 122796804 | *C5* | T/C | 0.07 | 0.24 | 6.28E-01 |
| rs3118470 | 10 | 6141719 | *IL2RA* | C/T | 0.27 | 0.23 | 6.28E-01 |
| rs4837805 | 9 | 122786076 | *C5* | G/A | 0.41 | 0.23 | 6.32E-01 |
| rs6540433 | 1 | 205720018 | *CR2* | C/A | 0.16 | 0.22 | 6.36E-01 |
| rs2104286 | 10 | 6139051 | *IL2RA* | G/A | 0.22 | 0.22 | 6.42E-01 |
| rs4425986 | 1 | 205360403 | *C4BPA* | C/T | 0.46 | 0.21 | 6.44E-01 |
| rs791589 | 10 | 6129577 | *IL2RA* | G/A | 0.18 | 0.20 | 6.51E-01 |
| rs6762462 | 3 | 188424749 | *MASP1* | A/C | 0.32 | 0.20 | 6.51E-01 |
| rs1833863 | 5 | 41125622 | intergenic | G/A | 0.29 | 0.20 | 6.52E-01 |
| rs12034383 | 1 | 205870218 | *CR1* | G/A | 0.41 | 0.20 | 6.55E-01 |
| rs2230205 | 19 | 6660704 | *C3* | A/G | 0.16 | 0.20 | 6.56E-01 |
| rs17375194 | 3 | 188470176 | *MASP1* | G/C | 0.13 | 0.20 | 6.57E-01 |
| rs11569450 | 19 | 6653455 | *C3* | G/C | 0.13 | 0.20 | 6.58E-01 |
| rs6772329 | 3 | 188397297 | *RTP1* | T/C | 0.11 | 0.19 | 6.60E-01 |
| rs629409 | 1 | 22859325 | *C1QB* | A/G | 0.19 | 0.19 | 6.60E-01 |
| rs12489890 | 3 | 188435109 | *MASP1* | A/G | 0.13 | 0.19 | 6.65E-01 |
| rs12107082 | 3 | 188439068 | *MASP1* | A/G | 0.06 | 0.19 | 6.66E-01 |
| rs831626 | 11 | 33702926 | *CD59* | G/A | 0.17 | 0.18 | 6.71E-01 |
| rs3087554 | 8 | 27511359 | *CLU* | G/A | 0.17 | 0.18 | 6.72E-01 |
| rs7842 | 12 | 8102454 | *C3AR1* | G/A | 0.29 | 0.18 | 6.75E-01 |
| rs992670 | 9 | 122821591 | *C5* | G/A | 0.44 | 0.18 | 6.75E-01 |
| rs11569538 | 19 | 6636723 | *C3* | C/G | 0.08 | 0.17 | 6.80E-01 |
| rs850317 | 3 | 188430495 | *MASP1* | A/G | 0.29 | 0.17 | 6.81E-01 |
| rs344555 | 19 | 6630360 | *C3* | A/G | 0.23 | 0.17 | 6.84E-01 |
| rs7968584 | 12 | 7042502 | *C1S* | T/C | 0.25 | 0.16 | 6.88E-01 |
| rs706780 | 10 | 6127032 | *IL2RA* | A/C | 0.05 | 0.16 | 6.88E-01 |
| rs1078375 | 5 | 41013517 | *C7* | T/C | 0.41 | 0.16 | 6.89E-01 |
| rs6680396 | 1 | 194899093 | *CFH* | G/A | 0.18 | 0.16 | 6.90E-01 |
| rs3792640 | 5 | 40968595 | *C7* | A/G | 0.21 | 0.16 | 6.91E-01 |
| rs706779 | 10 | 6138830 | *IL2RA* | G/A | 0.48 | 0.15 | 6.98E-01 |
| rs344546 | 19 | 6638132 | *C3* | T/C | 0.41 | 0.15 | 6.99E-01 |
| rs344542 | 19 | 6638517 | *C3* | C/T | 0.41 | 0.15 | 6.99E-01 |
| rs647571 | 1 | 57192195 | *C8B* | A/G | 0.34 | 0.15 | 7.01E-01 |
| rs17549193 | 9 | 136918847 | *FCN2* | T/C | 0.28 | 0.15 | 7.02E-01 |
| rs12153063 | 5 | 40943951 | *C7* | A/C | 0.10 | 0.15 | 7.02E-01 |
| rs3745568 | 19 | 6641613 | *C3* | C/A | 0.12 | 0.14 | 7.05E-01 |
| rs1061443 | 5 | 41018379 | *C7* | T/G | 0.23 | 0.14 | 7.06E-01 |
| rs3914010 | 3 | 188422267 | *MASP1* | G/A | 0.16 | 0.14 | 7.06E-01 |
| rs344550 | 19 | 6633953 | *C3* | G/C | 0.35 | 0.14 | 7.10E-01 |
| rs930507 | 10 | 54198272 | *MBL2* | G/C | 0.21 | 0.14 | 7.10E-01 |
| rs9998151 | 4 | 110886794 | *CFI* | C/T | 0.05 | 0.14 | 7.11E-01 |
| rs8112351 | 19 | 6657298 | *C3* | T/C | 0.24 | 0.14 | 7.11E-01 |
| rs12073436 | 1 | 22843495 | *C1QC* | T/C | 0.11 | 0.13 | 7.14E-01 |
| rs12116643 | 1 | 195239806 | *CFHR5* | C/T | 0.12 | 0.13 | 7.15E-01 |
| rs3813946 | 1 | 205694316 | *CR2* | G/A | 0.20 | 0.13 | 7.22E-01 |
| rs4151657 | 6 | 32025519 | *CFB* | C/T | 0.34 | 0.12 | 7.25E-01 |
| rs13157656 | 5 | 41000609 | *C7* | C/A | 0.22 | 0.12 | 7.28E-01 |
| rs3826945 | 19 | 813912 | *CFD* | G/A | 0.29 | 0.12 | 7.29E-01 |
| rs698085 | 3 | 188480117 | *MASP1* | C/T | 0.32 | 0.12 | 7.30E-01 |
| rs11595876 | 10 | 54196768 | *MBL2* | C/T | 0.08 | 0.11 | 7.35E-01 |
| rs9429944 | 1 | 205736930 | *CR1* | T/A | 0.22 | 0.11 | 7.35E-01 |
| rs6883180 | 5 | 41216128 | *C6* | C/T | 0.40 | 0.11 | 7.38E-01 |
| rs537160 | 6 | 32024379 | *CFB* | T/C | 0.23 | 0.11 | 7.38E-01 |
| rs406514 | 19 | 6653598 | *C3* | C/T | 0.25 | 0.11 | 7.44E-01 |
| rs7527218 | 1 | 205690452 | intergenic | G/A | 0.48 | 0.11 | 7.45E-01 |
| rs594661 | 1 | 57174570 | *C8B* | A/G | 0.08 | 0.10 | 7.48E-01 |
| rs6602392 | 10 | 6118085 | *IL2RA* | A/C | 0.13 | 0.10 | 7.48E-01 |
| rs2876849 | 5 | 41015547 | *C7* | T/A | 0.22 | 0.10 | 7.48E-01 |
| rs4915559 | 1 | 195153393 | *CFHR4* | C/T | 0.18 | 0.10 | 7.49E-01 |
| rs2279590 | 8 | 27512170 | *CLU* | A/G | 0.43 | 0.10 | 7.54E-01 |
| rs12139795 | 1 | 205697828 | *CR2* | G/A | 0.21 | 0.09 | 7.58E-01 |
| rs4317805 | 1 | 205719615 | *CR2* | A/G | 0.08 | 0.09 | 7.58E-01 |
| rs683916 | 1 | 57198062 | *C8B* | A/T | 0.32 | 0.09 | 7.59E-01 |
| rs187875 | 5 | 39351502 | *C9* | A/G | 0.34 | 0.09 | 7.64E-01 |
| rs4935047 | 10 | 54200073 | *MBL2* | A/G | 0.45 | 0.09 | 7.66E-01 |
| rs10922106 | 1 | 194958087 | *CFH* | G/A | 0.37 | 0.09 | 7.66E-01 |
| rs11064498 | 12 | 7041768 | *C1S* | G/A | 0.13 | 0.09 | 7.68E-01 |
| rs2511989 | 11 | 57134901 | *SERPING1* | A/G | 0.40 | 0.09 | 7.68E-01 |
| rs344548 | 19 | 6636817 | *C3* | C/G | 0.18 | 0.08 | 7.74E-01 |
| rs942201 | 10 | 6126298 | *IL2RA* | T/G | 0.19 | 0.08 | 7.77E-01 |
| rs2455308 | 5 | 40965270 | *C7* | T/C | 0.21 | 0.08 | 7.79E-01 |
| rs344543 | 19 | 6638414 | *C3* | C/G | 0.41 | 0.08 | 7.79E-01 |
| rs17301146 | 1 | 57172849 | *C8B* | A/G | 0.14 | 0.08 | 7.84E-01 |
| rs2072633 | 6 | 32027557 | *CFB* | T/C | 0.40 | 0.07 | 7.84E-01 |
| rs11120218 | 1 | 205345074 | *C4BPA* | A/G | 0.17 | 0.07 | 7.85E-01 |
| rs534663 | 5 | 39376318 | *C9* | T/C | 0.43 | 0.07 | 7.88E-01 |
| rs698104 | 3 | 188485211 | *MASP1* | T/C | 0.20 | 0.07 | 7.88E-01 |
| rs263275 | 5 | 39350934 | *C9* | T/G | 0.43 | 0.07 | 7.89E-01 |
| rs2002665 | 3 | 188434166 | *MASP1* | A/G | 0.29 | 0.07 | 7.91E-01 |
| rs11569523 | 19 | 6640042 | *C3* | A/G | 0.09 | 0.07 | 7.94E-01 |
| rs11256456 | 10 | 6120718 | *IL2RA* | C/T | 0.18 | 0.07 | 7.96E-01 |
| rs237554 | 19 | 6637659 | *C3* | A/G | 0.15 | 0.07 | 7.97E-01 |
| rs2298749 | 4 | 110900954 | *CFI* | A/G | 0.26 | 0.06 | 7.99E-01 |
| rs17220750 | 9 | 122827820 | *C5* | A/G | 0.10 | 0.06 | 8.00E-01 |
| rs17514136 | 9 | 136912485 | *FCN2* | G/A | 0.26 | 0.06 | 8.00E-01 |
| rs11256497 | 10 | 6127800 | *IL2RA* | A/G | 0.41 | 0.06 | 8.04E-01 |
| rs12722563 | 10 | 6109567 | *IL2RA* | T/C | 0.10 | 0.06 | 8.05E-01 |
| rs12756603 | 1 | 22854463 | *C1QB* | G/A | 0.16 | 0.06 | 8.06E-01 |
| rs10512766 | 5 | 41249092 | *C6* | G/C | 0.29 | 0.06 | 8.06E-01 |
| rs885448 | 4 | 110910108 | *CFI* | A/T | 0.24 | 0.06 | 8.06E-01 |
| rs2241394 | 19 | 6636230 | *C3* | G/C | 0.12 | 0.06 | 8.07E-01 |
| rs7899538 | 10 | 6099904 | *IL2RA* | A/C | 0.12 | 0.06 | 8.09E-01 |
| rs7524776 | 1 | 194889960 | *CFH* | C/T | 0.14 | 0.06 | 8.11E-01 |
| rs11256448 | 10 | 6119485 | *IL2RA* | G/A | 0.23 | 0.05 | 8.17E-01 |
| rs4403634 | 1 | 205334714 | *C4BPB* | A/C | 0.50 | 0.05 | 8.21E-01 |
| rs1444903 | 5 | 41240060 | *C6* | T/C | 0.14 | 0.05 | 8.24E-01 |
| rs2989726 | 9 | 136949195 | *FCN1* | G/A | 0.25 | 0.05 | 8.25E-01 |
| rs16840422 | 1 | 194919457 | *CFH* | T/C | 0.17 | 0.05 | 8.27E-01 |
| rs2228150 | 10 | 6107975 | *IL2RA* | A/G | 0.03 | 0.05 | 8.27E-01 |
| rs6696924 | 1 | 57132516 | *C8A* | G/T | 0.25 | 0.05 | 8.28E-01 |
| rs17020983 | 1 | 205354920 | *C4BPA* | T/C | 0.11 | 0.05 | 8.29E-01 |
| rs7024491 | 9 | 136914905 | *FCN2* | G/A | 0.39 | 0.05 | 8.31E-01 |
| rs7519492 | 1 | 195106748 | intergenic CFHR1-CFHR4 | C/T | 0.17 | 0.04 | 8.34E-01 |
| rs2250656 | 19 | 6669534 | *C3* | G/A | 0.24 | 0.04 | 8.37E-01 |
| rs850307 | 3 | 188464162 | *MASP1* | A/C | 0.27 | 0.04 | 8.37E-01 |
| rs2749812 | 20 | 23010927 | *CD93* | A/G | 0.13 | 0.04 | 8.40E-01 |
| rs850316 | 3 | 188430846 | *MASP1* | T/C | 0.41 | 0.04 | 8.43E-01 |
| rs3815623 | 3 | 188441736 | *MASP1* | T/C | 0.47 | 0.04 | 8.45E-01 |
| rs7041446 | 9 | 136915875 | *FCN2* | A/G | 0.49 | 0.04 | 8.47E-01 |
| rs16840522 | 1 | 194977539 | *CFH* | C/T | 0.21 | 0.03 | 8.54E-01 |
| rs6690215 | 1 | 205722673 | *CR2* | C/T | 0.45 | 0.03 | 8.56E-01 |
| rs12685659 | 9 | 136916540 | *FCN2* | T/A | 0.10 | 0.03 | 8.58E-01 |
| rs1418471 | 1 | 57131889 | *C8A* | G/A | 0.13 | 0.03 | 8.60E-01 |
| rs12658133 | 5 | 40968376 | *C7* | G/A | 0.20 | 0.03 | 8.60E-01 |
| rs324058 | 5 | 40997651 | *C7* | G/T | 0.24 | 0.03 | 8.61E-01 |
| rs876650 | 3 | 188434334 | *MASP1* | C/G | 0.04 | 0.03 | 8.62E-01 |
| rs2989735 | 9 | 136941237 | *FCN1* | T/C | 0.41 | 0.03 | 8.65E-01 |
| rs4469075 | 4 | 110910035 | *CFI* | C/G | 0.31 | 0.03 | 8.72E-01 |
| rs11861251 | 16 | 31196897 | *CD11B* | C/T | 0.15 | 0.03 | 8.74E-01 |
| rs6686359 | 1 | 57129168 | *C8A* | C/T | 0.04 | 0.02 | 8.76E-01 |
| rs3128627 | 9 | 136912613 | *FCN2* | G/A | 0.07 | 0.02 | 8.83E-01 |
| rs2416815 | 9 | 94472478 | intergenic | A/G | 0.07 | 0.02 | 8.83E-01 |
| rs17216529 | 9 | 122840039 | *C5* | A/G | 0.07 | 0.02 | 8.86E-01 |
| rs2236217 | 1 | 57203393 | *C8B* | T/G | 0.11 | 0.02 | 8.90E-01 |
| rs619545 | 1 | 57145179 | *C8A* | G/A | 0.28 | 0.02 | 8.91E-01 |
| rs17201103 | 5 | 41221403 | *C6* | T/C | 0.29 | 0.02 | 9.00E-01 |
| rs1468673 | 9 | 122849711 | *C5* | C/T | 0.37 | 0.01 | 9.03E-01 |
| rs1801033 | 5 | 41235716 | *C6* | C/A | 0.35 | 0.01 | 9.06E-01 |
| rs791590 | 10 | 6130328 | *IL2RA* | T/A | 0.12 | 0.01 | 9.07E-01 |
| rs7046 | 11 | 33683556 | *CD59* | T/C | 0.37 | 0.01 | 9.08E-01 |
| rs1143664 | 12 | 7045308 | *C1S* | G/A | 0.12 | 0.01 | 9.12E-01 |
| rs679350 | 1 | 57137400 | *C8A* | T/C | 0.22 | 0.01 | 9.16E-01 |
| rs10824794 | 10 | 54203195 | *MBL2* | T/A | 0.21 | 0.01 | 9.19E-01 |
| rs850313 | 3 | 188435920 | *MASP1* | G/C | 0.29 | 0.01 | 9.20E-01 |
| rs3792642 | 5 | 40968910 | *C7* | T/C | 0.17 | 0.01 | 9.20E-01 |
| rs2300932 | 9 | 22810257 | *C5* | A/C | 0.42 | 0.01 | 9.20E-01 |
| rs6674522 | 1 | 195182982 | *CFHR2* | C/G | 0.13 | 0.01 | 9.26E-01 |
| rs4521835 | 9 | 136919127 | *FCN2* | G/T | 0.43 | 0.01 | 9.26E-01 |
| rs2405237 | 1 | 57113134 | *C8A* | A/G | 0.03 | 0.01 | 9.29E-01 |
| rs344544 | 19 | 6638148 | *C3* | T/G | 0.15 | 0.01 | 9.30E-01 |
| rs2416810 | 9 | 122825021 | *C5* | A/G | 0.15 | 0.01 | 9.34E-01 |
| rs7072398 | 10 | 6119852 | *IL2RA* | A/G | 0.45 | 0.01 | 9.36E-01 |
| rs1576340 | 1 | 194965334 | *CFH* | A/C | 0.16 | 0.01 | 9.37E-01 |
| rs6602391 | 10 | 6118038 | *IL2RA* | A/G | 0.08 | 0.01 | 9.40E-01 |
| rs17186582 | 1 | 205352254 | *C4BPA* | A/C | 0.29 | 0.01 | 9.40E-01 |
| rs7135975 | 12 | 7134709 | *CIR* | G/A | 0.24 | 0.00 | 9.44E-01 |
| rs8942 | 1 | 205336542 | *C4BPB* | T/C | 0.21 | 0.00 | 9.45E-01 |
| rs344545 | 19 | 6638142 | *C3* | T/C | 0.41 | 0.00 | 9.49E-01 |
| rs11569514 | 19 | 6641276 | *C3* | T/A | 0.11 | 0.00 | 9.56E-01 |
| rs662377 | 1 | 57097100 | *C8A* | T/C | 0.42 | 0.00 | 9.61E-01 |
| rs17057444 | 8 | 27509710 | *CLU* | G/C | 0.05 | 0.00 | 9.61E-01 |
| rs17201075 | 5 | 41221364 | *C6* | A/G | 0.06 | 0.00 | 9.61E-01 |
| rs6699859 | 1 | 57133038 | *C8A* | T/C | 0.32 | 0.00 | 9.68E-01 |
| rs7027797 | 9 | 122811620 | *C5* | C/T | 0.08 | 0.00 | 9.69E-01 |
| rs3733003 | 3 | 188421885 | *MASP1* | A/T | 0.32 | 0.00 | 9.72E-01 |
| rs12377780 | 9 | 136948782 | *FCN1* | A/C | 0.32 | 0.00 | 9.72E-01 |
| rs638919 | 1 | 57149760 | *C8A* | G/A | 0.26 | 0.00 | 9.73E-01 |
| rs12106739 | 3 | 188473505 | *MASP1* | G/A | 0.09 | 0.00 | 9.78E-01 |
| rs1357134 | 3 | 188462462 | *MASP1* | G/A | 0.41 | 0.00 | 9.79E-01 |
| rs12685344 | 9 | 122804812 | *C5* | A/G | 0.07 | 0.00 | 9.80E-01 |
| rs17612 | 9 | 122765747 | *C5* | C/A | 0.05 | 0.00 | 9.84E-01 |
| rs710462 | 3 | 188445824 | *MASP1* | G/T | 0.50 | 0.00 | 9.85E-01 |
| rs7040603 | 9 | 122848041 | *C5* | T/C | 0.21 | 0.00 | 9.85E-01 |
| rs11720718 | 3 | 188424123 | *MASP1* | A/G | 0.32 | 0.00 | 9.88E-01 |
| rs12136082 | 1 | 11024862 | *MASP2* | A/G | 0.18 | 0.00 | 9.90E-01 |
| rs698084 | 3 | 188480342 | *MASP1* | A/G | 0.18 | 0.00 | 9.93E-01 |
| rs17021100 | 1 | 205360630 | *C4BPA* | C/G | 0.11 | 0.00 | 9.95E-01 |
| rs2241393 | 19 | 6636304 | *C3* | G/C | 0.37 | 0.00 | 1 |
